# The influence of the crowding assumptions in biofilm simulations

**DOI:** 10.1371/journal.pcbi.1009158

**Published:** 2021-07-22

**Authors:** Liliana Angeles-Martinez, Vassily Hatzimanikatis

**Affiliations:** Laboratory of Computational Systems Biotechnology, École Polytechnique Fédérale de Lausanne, EPFL, Lausanne, Switzerland; University of Cambridge, UNITED KINGDOM

## Abstract

Microorganisms are frequently organized into crowded structures that affect the nutrients diffusion. This reduction in metabolite diffusion could modify the microbial dynamics, meaning that computational methods for studying microbial systems need accurate ways to model the crowding conditions. We previously developed a computational framework, termed CROMICS, that incorporates the effect of the (time-dependent) crowding conditions on the spatio-temporal modeling of microbial communities, and we used it to demonstrate the crowding influence on the community dynamics. To further identify scenarios where crowding should be considered in microbial modeling, we herein applied and extended CROMICS to simulate several environmental conditions that could potentially boost or dampen the crowding influence in biofilms. We explore whether the nutrient supply (rich- or low-nutrient media), the cell-packing configuration (square or hexagonal spherical cell arrangement), or the cell growing conditions (planktonic state or biofilm) modify the crowding influence on the growth of *Escherichia coli*. Our results indicate that the growth rate, the abundance and appearance time of different cell phenotypes as well as the amount of by-products secreted to the medium are sensitive to some extent to the local crowding conditions in all scenarios tested, except in rich-nutrient media. Crowding conditions enhance the formation of nutrient gradient in biofilms, but its effect is only appreciated when cell metabolism is controlled by the nutrient limitation. Thus, as soon as biomass (and/or any other extracellular macromolecule) accumulates in a region, and cells occupy more than 14% of the volume fraction, the crowding effect must not be underestimated, as the microbial dynamics start to deviate from the *ideal/expected* behaviour that assumes volumeless cells or when a homogeneous (reduced) diffusion is applied in the simulation. The modeling and simulation of the interplay between the species diversity (cell shape and metabolism) and the environmental conditions (nutrient quality, crowding conditions) can help to design effective strategies for the optimization and control of microbial systems.

## Author summary

In nature, many organisms grow in crowded biofilms that protect against stressful conditions, making their control/eradication a challenge. Modeling these microbial systems is a valuable tool for studying the interactions among cells and exploring strategies for manipulating the system. Even though the composition of biofilms changes over time due to the accumulation of biomolecules in the medium as well as the growth of cells—both in size and number, many current modeling methods do not explicitly take into account for these changes. This study analyzes how sensitive the biofilm simulation is to these crowding conditions to determine whether they can be safely ignored or need to be included for accurate results. We compared different simplifications of the crowding effect on spatio-temporal microbial simulations under several scenarios. We found that the traditional use of a reduced diffusion constant fails to capture the heterogeneous nature of a biofilm and could introduce deviations to the dynamics of the system (biomass, phenotypes, metabolic production), especially in poor nutrient mediums. The crowding conditions modeling in microbial systems can provide a guidence for selecting effective treatments to disrupt and control biofilms associated to chronic diseases.

## Introduction

The spatio-temporal modeling of microbial systems can shed light on the dynamics and species interactions [1–4], the pattern formation [5–7] as well as the response of microbial communities to enviromental changes, e.g. the secretion and accumulation of weak acid products [8], the addition of new species to the system [1], the exposure to antibiotics [9] or to a nutrient shift [10]. Frequently, microbial communities are forced to grow in space constraints, where the proximity to cells and other solid components (proteins, DNA, polysaccharides) reduce the availability and diffusion of nutrients as well as the motility of the cells [11,12]. The crowding conditions (i.e. the volume fraction occupied by cells and macromolecules) change over time accentuating the heterogeneous nature of the system, where the spatial differences in the local availability of the nutrients affects the dynamics of the whole community. Although the crowding effect has been acknowledged in the microbial modeling, e.g. by reducing the nutrient diffusion, less attention has been paid on the impact of the crowding assumption/simplification on microbial simulations. We herein focused on this aspect, analyzing how the environmental conditions could increase/reduced the importance of the crowding assumptions on biofilm simulations.

Several frameworks have already been proposed to integrate the metabolic information of microbial species, estimated using either Monod kinetics [5–7,9] or techniques such as Flux Balance Analysis [1–4,8,10], and the spatial distribution of the nutrients in the system (computed from the diffusion equation). The ability of these models to successfully predict the behavior and interactions within microbial communities makes them a valuable tool for the study of complex ecosystems. Frequently, however, these models either neglect or over/underestimate the influence of the crowding conditions on microbial systems by assuming diluted systems or certain degree of homogeneity, e.g. using a reduced diffusion constant [4] or a constant cell volume [3]. This could lead to a miscalculation of the microbial behavior in crowded systems, such as in calculating the effects of antibiotics on treating a biofilm infection.

Recently, we developed **CRO**wding **M**odeling of **I**n-silico **C**ommunity **S**ystems (CROMICS), a methodology that captures the effect of crowding conditions on the spatio-temporal modeling of microbial communities (Manuscript submitted). CROMICS combines techniques such as individual-based modeling (IbM) and thermodynamics flux analysis (TFA) [13] to simulate the behavior of individual cells, where the metabolic capabilities of each species are estimated from the stoichiometry of the metabolic networks. Additionally, the scaled particle theory (SPT) [14,15] is used to model the effect of crowding on the effective diffusion coefficient and concentration of metabolites. In the original CROMICS version, cells and metabolites are allowed to move in a square lattice (or cubic grid for 3D systems), and also the metabolic fluxes estimation of each cell neglects any restriction imposed by the proteome availability to carry out the metabolic functions. Here, CROMICS has been extended to improve the cell metabolism predictions and study the effect of lattice geometries on the microbial simulations.

The cell has a finite proteome capacity, the way in which proteome resources are distributed determines the growth rate and the synthesis of by-products, e.g. under aerobic conditions fast growing *Escherichia coli* incompletely oxidizes glucose secreting acetate as way to optimize the limited enzymes available. In this paper, specifically, we implement a proteome allocation version of TFA (here identified aTFA) to ensure that the computed metabolic fluxes are given by (i) the optimal distribution of the cell proteome to carry out the cell growth and synthesis of enzymes [16], and (ii) in the direction of the Gibbs free energy drop [13]. CROMICS can simulate the diffusion of nutrients across a lattice-on system using a crowding Lattice Boltzmann (cLBM) scheme [17]. To explore the effect of different cellular arrangements, we have herein extended cLBM (originally implemented for a cubic grid) to simulate an additional lattice geometry: the hexagonal grid. Additionally, the IbM rules for the cell motion/disribution in the hexagonal grid system were also updated.

We showed previously that crowding conditions can modify the microbial competition (Manuscript submitted), and herein sought to systematically evaluate different crowding simplifications and environmental conditions (nutrient supply, spherical cell arrangement, and planktonic/biofilm cell growing conditions) to determine the scenarios in which the crowding conditions have a greater impact in biofilms. The simulation results of the *E*. *coli* growth indicated that the use of a (constant) reduced diffusion coefficient as a way of simplifying the crowding effect can alter the production of biomass and metabolic by-products by changing the local availability of nutrients compared to the distributions computed when the cell size is explicitly considered in the simulation. Only rich-nutrient media can dampen the crowding influence on the simulation. Overall, these results highlight the influence of the crowding conditions, even in microbial systems other than biofilms, and the importance of selecting of an appropriate crowding assumption depending of the characteristic of the system.

## Results and discussion

### Case study: Biofilm formation by *E*. *coli*

To investigate the influence of crowding conditions on the microbial growth of clustered systems, we simulated the aerobic growth of *E*. *coli* iJ01366 [18] on glucose in a 2D system. The system consists of a monolayer of cubic boxes of Δ*x* per side, which can allocate up to 10,000 spherical cells of maximum radius *R*_*max*,*cell*_. The cell volume is assumed to be proportional to the cell mass *M*_*cell*_ and the specific volume constant *υ*_*sp*_, i.e. $4\pi R_{cell}^{3}/3=M_{cell}\upsilon_{sp}$. Thus, *R*_*max*,*cell*_ was computed based on the maximal cell dry mass *M*_*max*,*sp*_ as *R*_*max*,*cell*_ = (3*M*_*max*,*sp*_*υ*_*sp*_/4*π*)^1/3^ (Eq 22). All of the parameters and constants for the system are given in Table 1. To test this system, an initial seed of 820 cells were randomly allocated at the bottom of the system. The mass of each cell was randomly taken from a normal distribution, with a mean of 4.89 × 10^−13^ g_DW_ and a standard deviation of 1.32 × 10^−13^ g_DW_ [19]. Unless otherwise stated, the cells were considered to be attached to the surface and/or to the biofilm, meaning they can only move to a neighboring box by cell shoving. Bounceback boundaries were set for both cells and metabolites, meaning they were not allowed to leave the system.

**Table 1:** Parameters used in CROMICS simulations.

Parameter	Description	Value	Units	Ref.
*D*_*w*,*glucose*_	Glucose diffusion in water	6.7 x 10^−7^	mm^2^ ms^-1^	[12]
*D*_*w*,*oxygen*_	Oxygen diffusion in water	2 x 10^−6^	mm^2^ ms^-1^	[12]
*D*_*w*,*acetate*_	Acetate diffusion in water	1.21 x 10^−6^	mm^2^ ms^-1^	[12]
*D*_*sp*_	Diffusion of non-motile *E*. *coli*	2 x 10^−10^	mm^2^ ms^-1^	[20]
*V*_*M*,*glucose*_	Maximum glucose uptake rate	10	mmol g_DW_^-1^ h^-1^	[21]
*K*_*M*,*glucose*_	Michaelis constant for glucose	1.5 x 10^−2^	mM	[21]
*V*_*M*,*oxygen*_	Maximum oxygen uptake rate	15	mmol g_DW_^-1^ h^-1^	[21]
*V*_*M*,*acetate*_	Maximum acetate uptake rate	17	mmol g_DW_^-1^ h^-1^	[22]
*υ*_*sp*_	Cell specific volume	3.07 x 10^3^	mm^3^ g_DW_^-1^	a
*υ*_*met*_	Metabolite specific volume	7.3 x 10^2^	mm^3^ g^-1^	[23]
*M*_*min*,*sp*_	Minimal dry mass of *E*. *coli*	8.3 x 10^−14^	g_DW_	[19]
*M*_*max*,*sp*_	Maximal dry mass of *E*. *coli*	1.172 x 10^−12^	g_DW_	[19]
*MW*_*protein*_	Protein molecular weight in the intracellular space	7.2 x 10^4^	Da	[23]
*v*_*shrinkage*_	Cell shrinkage rate	1.6 x 10^−2^	h^-1^	[24]

^a^ Computed as *υ*_*sp*_ = *ρ*_*sp*_^-1^ * *M*_*cell*,*wet*_/ *M*_*cell*,*dry*_, where *ρ*_*sp*_ = 1.105 g mL [25], *M*_*cell*,*dry*_ = 2.8 x 10^−13^ g_DW_, and *M*_*cell*,*wet*_ = 9.5 x 10^−13^ g [26].

As with other spatio-temporal models, the accuracy of the results computed by CROMICS depends on the parameters Δ*x* and Δ*t*. Here, we used a fine discretization of the system where a maximum of one cell is allowed per box, thus Δ*x* = 2*R*_*max*,*cell*_ = 1.3 x 10^−3^ mm. To avoid numerical instabilities, Δ*t* was set to be proportional to the diffusion of the fastest metabolite in the system. In this case, it was O_2_, so Δ*t* = Δ*x*^*2*^/4*D*_*O2*_ = 0.2 ms.

To select the amount of glucose supplied to the system in the simulation, we considered that *E*. *coli* is found at the end of the small intestine where the glucose concentration is about 2.25 mM [27]. Here, we assumed that an infinite nutrient reservoir was located at the top boundary, with a constant concentration of 2.25 mM of glucose and 0.21 mM of O_2_ (only glucose and O_2_ can leave/enter the system through the top boundary). The O_2_ concentration (0.21 mM) at the top boundary was computed using Henry’s law, assuming that the fresh medium supplied was at equilibrium with a gas phase where the partial pressure of O_2_ was 0.162 atm, and with Henry’s law constant of 1.3 mmol L^−1^ atm^−1^ [28]. At time *t* = 0 h, all boxes with available space contained medium with 0.5 mM of glucose and 0.21 mM of O_2_. Since significant amounts of acetate are secreted under both aerobic and anaerobic conditions and in order to keep the biofilm problem as simple as possible, only the diffusion of glucose, O_2_, and acetate were tracked during the simulation. Because we assumed that cell radius *R*_*cell*_ was proportional to $M_{cell}^{1/3}$, the crowding conditions changed over time (this crowding assumption is identified as C1, see Table 2). The radii of the metabolites glucose, O_2_, and acetate were fixed to zero. All results were averaged over three replicate simulations.

**Table 2:** Crowding assumptions tested in the simulation.

Assumption	Description	Ref.
$\begin{array}{l} C1:\;{R_{cell}(t)\propto M_{cell}(t)}^{\frac{1}{3}}\\ D_{eff,met}=\gamma_{met}^{-1}D_{met} \end{array}$	**Time-dependent crowding** conditions. Cell radius *R*_*cell*_ is proportional to $M_{cell}^{1/3}$ (Eq 22).	[29]
C2: *R*_*cell*_ = 6.2 x 10^−4^ mm $D_{eff,met}=\gamma_{met}^{-1}D_{met}$	**Uniform crowding** conditions inside the biofilm. All cells have the same (average) volume (i.e. *R*_*cell*_ is constant).	[3,10]
C3: *R*_*cell*_ = 0 *D*_*eff*,*met*_ = 0.25*D*_*met*_	**Crowding** conditions are **neglected,** but the **metabolite diffusion** is **reduced**. A constant *D*_*eff*_ is applied in the entire system (i.e. in boxes with/without cells). Cells are volumeless.	[4,8,12]
C4: *R*_*cell*_ = 0 *D*_*eff*,*met*_ = *D*_*met*_	**Crowding** conditions are **neglected**. Cells are volumeless, and *D*_*eff*_ is equal to the metabolite diffusion in water.	[1,4,5,24]

**Fig 1:**
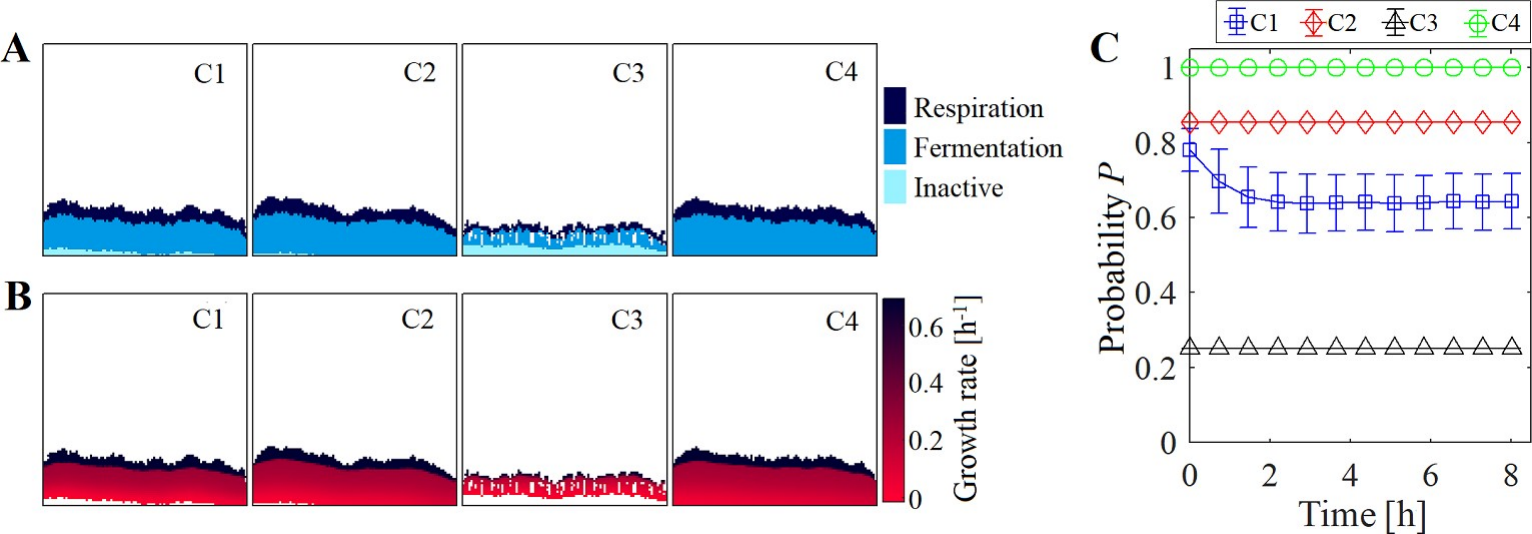
Formation of the *E*. *coli* biofilm with a glucose supply of 2.25 mM using a square lattice. (A) Phenotype distribution and (B) growth-rate gradients predicted for the *E*. *coli* biofilm at 3.7 h under the crowding assumptions C1-4 (Table 2). Phenotypes identified were respiration: glucose + O_2_ ➔ (acetate) + biomass, fermentation: glucose ➔ acetate + biomass, and inactive cells. Some cavities were observed inside the biofilm in C3 simulations due to the slow growth rate of the cells and the stochastic movements during the shoving process. (C) Probability *P* to find available space for glucose diffusion in biofilm-boxes computed at different times. Error bars show the standard deviation in three independent simulations.

Although the system started with a homogeneous distribution of substrates, the microbial activity rapidly formed a nutrient gradient inside and above the biofilm (S1 Fig), which caused different phenotypes to emerge. One of these phenotypes is based on the ability of *E*. *coli* to metabolize glucose under different oxygenation conditions. Since the amount of O_2_ supplied to the system was limited (0.21 mM), the cells growing aerobically were only located in the superficial layers of the biofilm (respiration phenotype: glucose + O_2_ ➔ acetate + biomass) (see C1 in Fig 1A). In the presence of O_2_, ATP is obtained from respiratory pathways, where the glucose is oxidized into CO_2_. However, fast-growing cells (*v*_*bio*_ ≥ 0.4 h^-1^) opt for an overflow metabolism that produces acetate even if O_2_ is available in the medium [16]. This cellular strategy assigns a higher fraction of the proteome to the synthesis of biomass building blocks at the expense of energy production. Thus, energy biogenesis follows a fermentative pathway, which is ~50% more protein-efficient than the respiratory one [30]. The allocation constraint [16] included in aTFA allows to capture the overflow metabolism without any ad hoc restriction that prevents the acetate secretion in cells with *v*_*bio*_ < 0.4 h^-1^. When glucose is still abundant, the optimal proteome allocation also prevented the (unrealistic) simultaneous consumption of glucose and acetate [31]. This restriction is due to the fact that to produce the same amount of biomass, the cell has to consume larger amounts acetate, therefore more enzymes are required for the acetate catabolism, compared to the glucose necessary (and the corresponding enzymes). In other words, the cell prefers (from the proteome point of view) to use glucose than acetate. Nevertheless, recent studies showed that *E*. *coli* can consume both substrates simultaneously when the extracellular acetate concentration is high [32]. In our simulations, the sequence of substrate uptake was determined by the proteome allocation of the cells (using aTFA), and not by the imposition of zero acetate uptake flux until glucose has been depleted.

A fermentation phenotype (glucose ➔ acetate + biomass) was identified at the bottom and middle regions of the system where O_2_ was already depleted but where glucose was still available (C1 in Fig 1A). In such conditions, *E*. *coli* shifts the metabolism to a fermentative one that synthesizes acetate, ethanol, and formate as the main by-products of glucose [33]. Even cells of the same phenotype achieved different growth rate due to the nutrients gradient formed in the biofilm (C1 in Fig 1B). As the simulation progressed, regions with inactive cells appeared at the bottom of the biofilm (C1 in Fig 1A) in regions where the glucose supply was completely exhausted. In light of this, snapshots of the spatial distribution of metabolites and phenotypes predicted at different times under C1 are shown S1 Fig. The phenotypic differentiation of species resulting from the cell adaptation to local enviromental conditions has been experimentally observed in biofilms [34], where fast-growing cells were identified at the top of the biofilm wherein O_2_ is availiable, while at the bottom, the anoxic regions are characterized by dormant or slow-growing cells. Although not considered in this case study, the CO_2_ produced during the glucose oxidation could reduce the partial pressure of O_2_ in the gas phase at equilibrium with the medium supplied, reducing in this way the concentration of O_2_ dissolved (computed by the Henry’s law) that can enter to the system through the top boundary. Further studies are required to determine the effect of CO_2_ production on the microbial dynamics. Overall, these results showed that the spatio-temporal models can reproduce the metabolic heterogeneity arising from the nutrients gradient formed in biofilms. In the following section, we investigated how different representations of the crowding effect in the microbial model modify the dynamics of the system.

### Effect of the crowding assumptions

The crowding conditions are given by the volume fraction occupied by the cells and other biofilm components (i.e. *V*_*occ*_/*V*_*tot*_). The presence of cells reduces the available volume for the motion and reaction of solutes in a biofilm. In this sense, macromolecular crowding reduces the replenishment of glucose and O_2_ inside the biofilm, and therefore modifies the behavior of the population. Often, this crowding effect has been considered in biofilm simulations by assuming a reduced and constant diffusion coefficient. However, the diffusion coefficient is a function of the local composition of the biofilm [11]. In this section, we analyze how different crowding assumptions could affect the biofilm simulations. For comparison purposes, four different crowding assumptions were tested (Table 2). In the first assumption (C1), a more detailed description of the time-dependent local crowding conditions was explicitly incorporated in the simulation by tracking the radii of the cells *R*_*cell*_, at every time point, as described in the Methods section. In the second assumption (C2), all cells had the same volume of 10^−9^ mm^3^, thus *R*_*cell*_ was constant and equal to 6.2 x 10^−4^ mm [3]. In the third assumption (C3), the diffusion was fixed at *D*_*eff*_ = 0.25*D* for all metabolites [12], and in the fourth assumption (C4), the crowding conditions were completely neglected, i.e. *D*_*eff*_ = *D*. In both C3 and C4, the cells were considered volumeless during the diffusion process, though overlapping cells were prohibited in the microbial spatial distributions.

**Fig 2:**
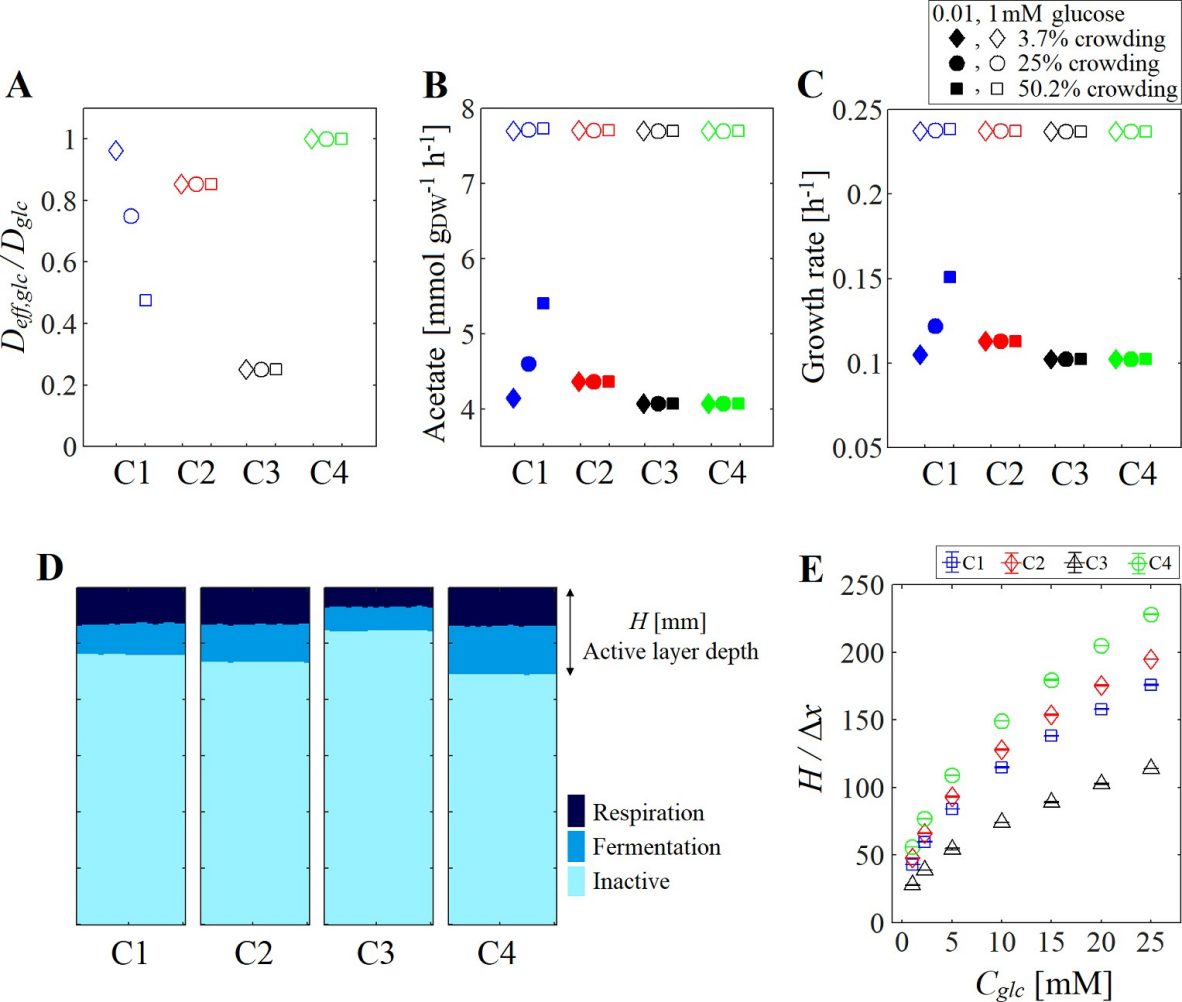
Diffusion and metabolic activity under different crowding assumptions. (A) Relative diffusion of glucose, (B) acetate flux and (C) growth rate predicted for a single cell in a box of 6.86 x 10^−9^ mm. Three crowding conditions were tested: 3.7% (using *R*_*cell*_ = 4 x 10^−4^ mm, diamond), 25% (*R*_*cell*_ = 7.4 x 10^−4^ mm, circle) and 50.2% (*R*_*cell*_ = 9.5 x 10^−4^ mm, square), and two different glucose concentration (*C*_*glc*_) were supplied: 0.01 mM (closed symbols) and 1mM (open symbols). The relative diffusion was not affected by *C*_*glc*_, therefore open and closed symbols overlap in (A). (D) Phenotype distribution predicted after 0.1 h for a small biofilm system under assumption C1-4 (Table 2). A squared lattice (Δ*x* = 1.3 x 10^−3^ mm) was used to simulate a system of 0.57 mm by 0.0380 mm filled with 6 000 *E*. *coli* cells, where glucose 2.25 mM was supplied from the top boundary. The simulation parameters are in Table 1. (E) Active layer depth *H* (normalized by Δ*x*) estimated in C1-4 for different *C*_*glc*_ supplied to system.

The activity coefficient of metabolite *met γ*_*met*_ (i.e. the ratio between the total volume and the available volume, *γ* = *V*_*tot*_/*V*_*av*_) can be used as an indicator of two opposing effects of the crowding conditions. *γ*_*met*_ can be computed as a function of the cell radii using SPT (Eq 17). On one hand, if the available volume decreases due to the presence of cells, that is *γ*_*met*_>1, the effective diffusion of *met* $D_{eff,met}=\gamma_{met}^{-1}D_{met}$ (Eq 25) decreases, but on the other hand, its effective concentration (i.e. the amount of *met* per available volume) *C*_*eff*,*met*_ = *C*_*met*_*γ*_*met*_ (Eq 15) increased. The overall outcome of these two aspects determines the nutrients availability in the system, and thus the microbial dynamics. To test how the different crowding assumptions can affect the diffusion and the effective concentration separately, we computed the relative diffusion of glucose (with radius *R*_*glc*_ = 0) and the anaerobic growth rate an *E*. *coli* cell in a box (6.86 x 10^−9^ mm^3^) with constant glucose concentration (i.e. mmol per total volume) *C*_*glc*_ = 0.01 mM under C1-4. Three cell radii *R*_*cell*_ were tested 4 x 10^−4^ mm (corresponding to the minimal cell dry mass), 7.4 x 10^−4^ mm, and 9.5 x 10^−4^ mm (corresponding to the maximal cell dry mass), so the volume fraction occupied in the box (i.e. the crowding conditions) were 3.7%, 25% and 50.2%, respectively. Results showed that the relative diffusion *D*_*eff*,*glc*_/*D*_*glc*_ decreased as the crowding increased only in C1, while in C2-C4 the relative diffusion remained constant independent on the crowding conditions (Fig 2A). Although SPT was used in C1 and 2 to compute the *γ*_*glc*_ (Eq 17), the relative diffusion in C2 remain constant because it was assumed a constant cell radius. Moreover, the increase of *γ*_*glc*_ in C1 due to the crowding conditions also enhanced the effective glucose concentration in the box (*C*_*eff*,*glc*_ = *C*_*glc*_*γ*_*glc*_) and with this the glucose uptake (given by the Michaelis-Menten equation). Thus, cell reached higher growth rate and acetate production rate as the crowding increased in C1 (Fig 2B and 2C). However, since the cell radius was considered constant in C2-4 (Table 2), the *γ*_*glc*_ computed by SPT was constant too, and the metabolic fluxes estimated were unaffected by increaments in the crowding conditions. We found that the difference in the metabolic fluxes computed in C1-4 (for all crowding conditions) decreased when the glucose concentration increased (*C*_*glc*_ = 1mM) (Fig 2B and 2C), that is the crowding effect became important under nutrient-limited conditions (as shown in the next section). In C3, the crowding effect was partially taken into account by assuming a reduced diffusion, but the effect was neglected on the metabolic activity by setting *R*_*cell*_ = 0, therefore *γ*_*glc*_ = 1 as in the assumption C4. In this example, the observed increase in the metabolic activity of the cell due to the crowding effect was possible because the amount of glucose in the box (i.e. the concentration *C*_*glc*_) was assumed constant. However, in biofilms, the replenishment of the glucose consumed depends on the diffusion rate that decreases with the crowding, so the access to nutrients is limited and, therefore, the metabolic activity of cells as well as the depth of the active layer in the biofilm can be reduced in such crowded environments.

To investigate how the depth of the active layer (i.e. region with cell activity) in the biofilm can be affected by the crowding assumptions made in the simulation, a small system of 0.57 mm by 0.0380 mm filled with 6,000 *E*. *coli* cells was simulated for 0.1 h (enough time to form the nutrient gradient, but without cell division). The same simulation parameters were used as in our base case study (Table 1). Unlike our base case study (described above) where nutrient gradients are formed also above the biofilm, and therefore the nutrients available for the surface cells depends on the distance from the top boundary at time *t*, in this example the surface cells are exposed to a constant nutrient concentration. Under all assumptions C1-4, three phenotypes were identified: respiration, fermentation and inactive cells appeared at the top, middle and bottom of the system, respectively (Fig 2D). However, the active layer depth *H* (Fig 2D), region dominated by respiration and fermentation phenotypes, depended on nutrient diffusion estimated in each crowding assumption. When the effective diffusion increased, the glucose was able to penetrate deeper in the biofilm before being depleted by cells (Fig 2E), that is *H*_*C3*_ (*D*_*eff*,*glc*_ = 1.6 x 10^−7^ mm^2^ ms^-1^) < *H*_*C1*_ (*D*_*eff*,*glc*_ = 5.1 x 10^−7^ ± 4 x 10^−8^ mm^2^ ms^-1^) < *H*_*C2*_ (*D*_*eff*,*glc*_ = 5.7 x 10^−7^ mm^2^ ms^-1^) < *H*_*C4*_ (*D*_*eff*,*glc*_ = 6.7 x 10^−7^ mm^2^ ms^-1^). Even more, when the glucose supply increased *H* increased too in C1-4 (Fig 2E). In general, the explicit simulation of the crowding conditions (C1) predicted a smaller *H* than ideal case C4. A small *H* (i.e. thin active layer) has been associated to strong segregation of microbial species in biofilms [5]. In this sense, the reduction of the available space due to the cell presence could also contribute to some extend to the emerging biofilm structure.

**Fig 3:**
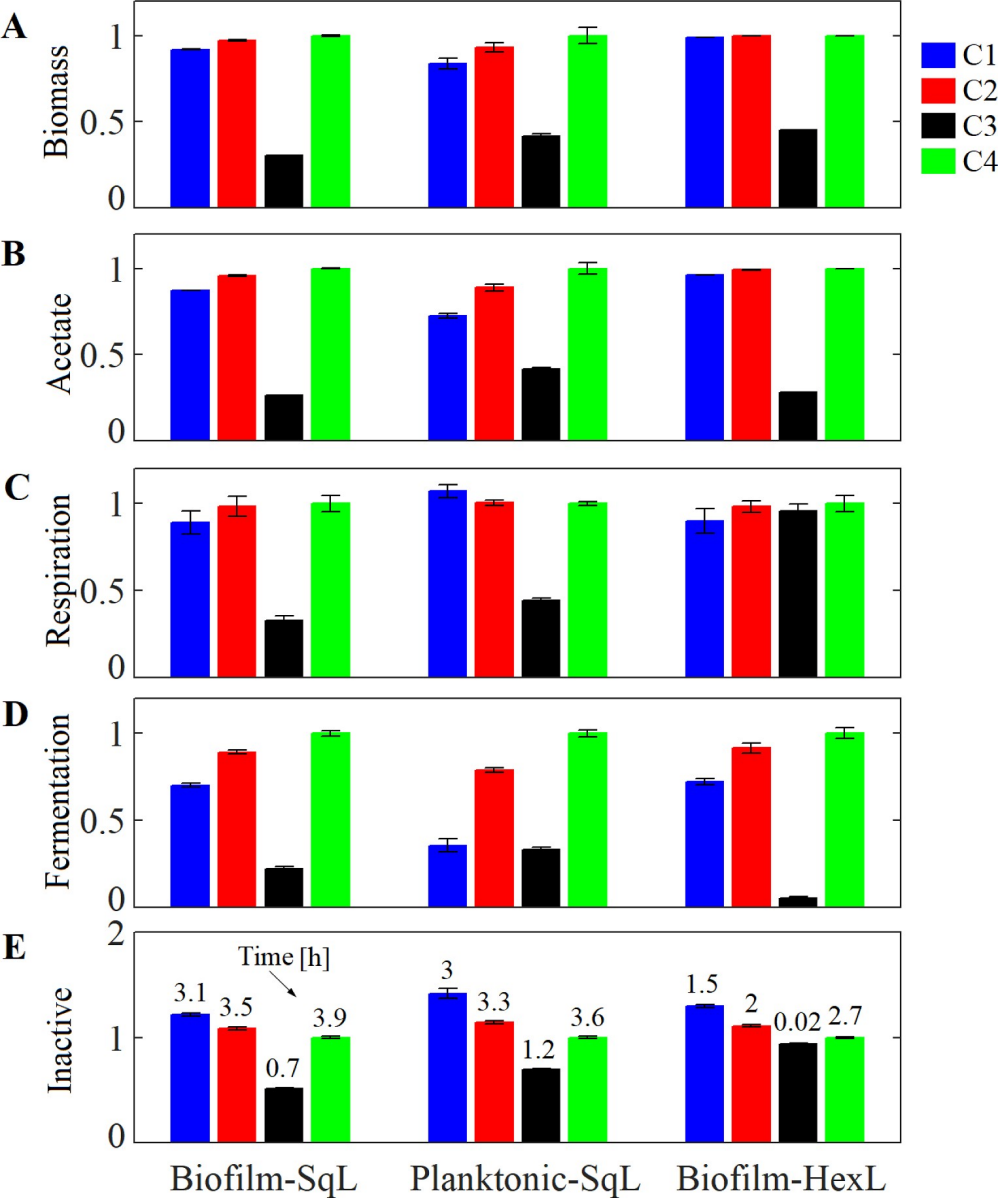
*E*. *coli* biofilm development at time 8.5 h with a glucose supply of 2.25 mM under different crowding assumptions. Three growth conditions and cell-packing configurations were tested: the biofilm growth using either a square lattice (Biofilm-SqL) or a hexagonal grid (Biofilm-HexL), and the planktonic mode of growth using a square lattice (Planktonic-SqL). (A) Relative biomass computed as Biomass(obtained in each C)/Biomass(obtained in C4) at time 8.5 h. (B) Relative acetate computed as Acetate(produced in each C)/Acetate(in C4). Relative number of cells identified with phenotype (C) respiration: glucose + O_2_ ➔ (acetate) + biomass, (D) fermentation: glucose ➔ acetate + biomass, and (E) inactive cells in each C compared to those obtained in C4. The number showed on bar plot (E) indicate the time in [h] when inactive cells appeared in the system. Crowding assumptions C1-4 are described in Table 2. Error bars show the standard deviation in three independent simulations.

Furthermore, to investigate the influence of the crowding assumptions in the biofilm development, we simulated the *E*. *coli* growth in a system with a glucose supply of 2.25 mM (our base case study). If the crowding conditions were explicitly incorporated in the simulation (i.e., C1), only a fraction of the system volume was unoccupied by cells so that the amount of nutrients that could be supplied to the system will be reduced due to space limitations. With less nutrients available in the system, cells changed/reduced their metabolism and, thus, the total biomass and acetate predicted at time 8.5 h under C1 was lower than when the crowding effect was completely neglected (C4) (Biofilm-SqL in Fig 3A and 3B). Although assumptions C2 and C3 integrated the crowding effect in the simulations, they did not provide significant improvements in the accuracy of the predictions for biomass and acetate compared to those obtained with C4. In C2, the assumption of a constant cell size captured the influence of the position and abundance of the individuals on the local crowding conditions, but the volume fraction occupied by the cells was only about 43% of that predicted by C1 at *t* = 8.5 h (S2 Fig). This underestimation of the volume occupied by cells under C2, and therefore the overestimation of the available volume, allowed more nutrients to penetrate the biofilm, and therefore more biomass and acetate were produced compared to C1. Reducing the crowding modeling complexity, in the case of C3, the entire system is considered homogeneous because the presence of cells did not affect the metabolites motion, instead a constant reduced diffusion was applied. Under such conditions, the glucose and O_2_ coming from the top of the system took about 4 times longer to get to the biofilm, which delayed the nutrient replenishment and in consequence the production of biomass and acetate was lower compared to other Cs (Biofilm-SqL in Fig 3A and 3B).

The different assumptions changed the number of active cells (respiration and fermentation phenotypes) that appeared in each simulation. In general, the number of active cells predicted for C4 was greater than the estimate for C1–3 (Biofilm-SqL in Fig 3C and 3D), likely because the cells represented no obstacle for the metabolites, so more O_2_ and glucose could enter in the system and penetrate deeper into the biofilm—leading to more acetate and biomass production (Biofilm-SqL in Fig 3A and 3B). In fact, the comparison of the spatial distribution of the phenotypes at 3.7 h showed regions with inactive cells at the bottom of the system for conditions C1–3, but not in C4, where inactive cells appeared after 3.9 h (Fig 1A). The delay in the emergence of inactice cells, the abundance of respiration and fermentation phenotypes as well as the biomass and acetate produced were associated to the ease of nutrients to diffuse inside the biofilm, which was determined by the crowding assumption made in the simulation (Biofilm-SqL in Fig 3). The deviations among the biomass and acetate estimated in C1, 2 and 4 started around the time inactive cells appeared in C1 (3.1 h) and increased over time (S3 Fig). The active layer depth at 3.1 h was ~30% of *H*_*C1*_ predicted when surface cells were exposed to a constant glucose concentration of 2.25 mM (as in the example shown in Fig 2E). In our base case study, the active layer depth (*H*) increased over time in C1-4 (S3 Fig). This is because during the biofilm expansion, the cells were pushed up to rich-nutrient regions near the top boundary (with 2.25mM of glucose and 0.21 mM of O_2_), thus, the amount of nutrients that penetrate in the biofilm increased over time.

The standard assumption in current methods for modeling crowding conditions involves fixing the diffusion rate as *D*_*eff*_ = 0.25*D*. However, when the constraint *D*_*eff*_ = 0.25*D* is applied over the whole system, this assumption (C3) provided the poorest approximation of the crowding effect compared to the simulations where the explicit crowding conditions were considered (C1). In C3, the cell metabolism was slowed down due to the nutrients scarcity caused by the slow re-supply of nutrients coming from the top boundary (as mentioned above). However, the reduction of metabolite diffusion due to the crowding effect is expected only in regions with cells and macromolecules (i.e. biofilm-boxes), which can be captured by assumption C2. We can show this by considering that in C2 cells occupy a constant volume in the box, which is similar to assuming crowding conditions where the reduced *D*_*eff*_ is only applied in boxes occupied by bacteria. In C2, the motion of metabolites from one box to another is limited by the probability *P* of finding available space, i.e. *D*_*eff*_ = *PD*, which is the key consideration on which cLBM is built [17]. For a volumeless metabolite, *P* = *γ*_*met*_^*-1*^ is equal to the volume fraction not occupied by cells (Eq 16). Thus, if we assume that cells occupy the 75% of the box at all times, then *P* is a constant value equal to 0.25, and C3 will behave similarly to C2.

From all assumptions tested, C2 provided the closest approximation to the more crowding detailed C1, though its overestimation of *P* caused that cells were metabolically more active. Based on the parameters selected in C2 for the average cell radius (*R*_*cell*_) and the box size (Δ*x*), *P* was computed equal to 0.85 for glucose, a value well above than the (average) *P* computed under C1 (Fig 1C). The probability *P* remained relatively constant after 2 h (*P* ~ 0.64) in C1, suggesting that an adjustment in the *R*_*cell*_ constant used in C2 (so that *P* of C2 matches *P* of C1, i.e. *R*_*cell*_ = 9.5 x 10^−4^ mm) could improve the accuracy of the C2 simulations of single species biofilm compared to those computed by C1.

From above, the crowding effect of C1 and C3 can be represented by C2 when changing the probability *P*. The difference between the probability used in C3 (*P* = 0.25) and the average value computed in C1 (*P* = 0.64) is likely because C1 only considers the effect crowding conditions, while the experimental observations used to fit *P* in C3 [12] also include the contribution of the electrostatic interactions, increment of the viscosity of the medium, etc. However, the simulation of more complex systems such as multi-species biofilms could require the more detailed crowding assumption C1 or the use of more parameters (e.g. different *R*_*cell*_ for each species) to account that the diffusion depends on the biofilm composition as was experimentally observed [11]. C1 can be further expanded to incorporate other type of interactions among the biofilm components that could affect the nutrients diffusion (e.g. electrostactic interactions), and in this way improve the accuracy of microbial predictions in structured and highly heterogeneous systems.

Additionally, for all crowding assumptions, a differentiated growth rate was also seen among the populations due to the gradient of the nutrient concentration formed in the biofilm (Fig 1B). This estimation of the local growth rate is very useful for antimicrobial studies, where the efficacy of a treatment depends on the physiology of the microorganism. For example, ribosome-targeting antibiotics inhibit ribosomal functions, such as translation [35]. Due to the positive correlation between the ribosome content and the growth rate [35], fast-growing cells are especially susceptible to this type of antibiotic. The crowding assumption modified the abundance of fast-growing cells in the system, e.g. cells with respiration phenotype can reach a maximum growth rate of 0.66 h^-1^, while the fermentation one only 0.27 h^-1^ (Figs 1B and 3C and 3D). In this sense, the accurate simulation of the crowding conditions could become important in the design of strategies for biofilm control (e.g. antimicrobial dose and/or exposure time) inasmuch as different growth-rate gradients could lead to ineffective or suboptimal antimicrobial treatments.

### Effect of the nutrient supply

The nutrient availability determines the cell metabolism, and thus the dynamics of the microbial community. Frequently, the amount of nutrients supplied to the system changes over time, such as when growing bacterial colonies on agar plates, where the carbon source has a finite initial concentration. In other cases, the nutrients supply is a function of spatial location, such as the glucose concentration variation from 0.2 to 50 mM along the intestine [27].

Even if crowding conditions hinder the diffusion of the metabolites, the exposure of a microbial community to a more nutrient-rich medium could dampen this crowding effect. Therefore, we analyzed the sensitivity of the microbial dynamics to the crowding conditions when different glucose concentrations were constantly supplied at the top boundary of the system. For this purpose, three different glucose concentrations were tested in our case study: 1.1, 10, and 25 mM, and we compared the results computed under the crowding assumptions C1–4 described in the previous section. In all cases, the initial glucose concentration in all boxes was fixed to 0.5 mM, while the top boundary glucose concentration was set to any of glucose supply values tested.

**Fig 4:**
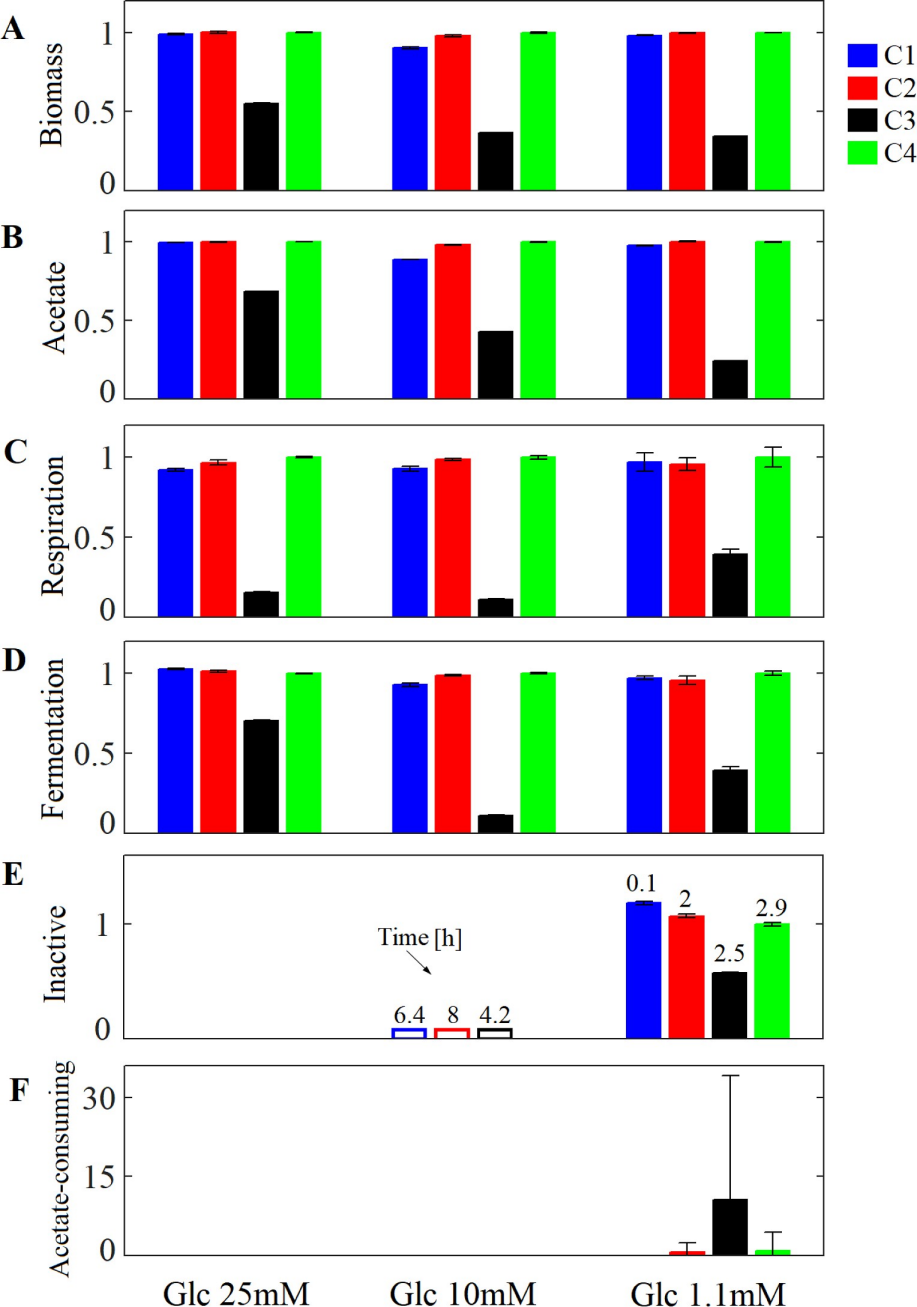
*E*. *coli* biofilm development at time 8.5 h with different glucose supplies and crowding assumptions using a square lattice. Three glucose supply (Glc) were tested: 25 mM, 10 mM and 1.1 mM. (A) Relative biomass computed as Biomass(obtained in each C)/Biomass(obtained in C4) at time 8.5 h. (B) Relative acetate computed as Acetate(produced in each C)/Acetate(in C4). Relative number of cells identified with phenotype (C) respiration: glucose + O_2_ ➔ (acetate) + biomass, (D) fermentation: glucose ➔ acetate + biomass, (E) inactive cells, and (F) acetate consuming: acetate + glucose + O_2_ ➔ biomass in each C compared to those obtained in C4. The number showed on bar plot (E) indicate the time in [h] when inactive cells appeared in the system. Inactive cells were not identified in C4 when glucose supply was set to 10mM. Crowding assumptions C1-4 are described in Table 2. Error bars show the standard deviation in three independent simulations.

As starting point, we simulated the growth of *E*. *coli* in a system with a high glucose supply of 25 mM. With this excess glucose, the biofilm grew rapidly, and during the first 8.5 h, the differences among the biomass and acetate estimated by the crowding assumptions C1, C2, and C4 were negligible (Fig 4). This is likely because in these three scenarios the cells were not able to metabolize all the glucose locally available, even if they worked at their maximum biological capacity. In other words, microbial dynamics was sensitive to the crowding conditions (given by C1 and 2) only when the nutrient gradient was formed and the cell metabolism was controlled by nutrient limitations. Even more, only active cells (respiration and fermentation phenotypes) were identified in the system under C1–4, and these phenotypes were present in a similar abundance in all cases. However, for C3 (with a reduced diffusion), the glucose and O_2_ supplied from the top of the system moves slowly through the biofilm, reducing the synthesized acetate and biomass compared to the other crowding assumptions. Consequently, inactive regions appeared only in C3 at the end of the simulation (Fig 4).

For an intermediate glucose supply of 10 mM, the system behaves similarly under the different crowding assumptions as the originally computed glucose concentration of 2.25 mM in the previous section, i.e., C4 and C2 overestimate the biomass production, while C3 underestimates the metabolic activity of the cells (Fig 4). This is likely because in both cases (glucose supply of 10 and 2.25 mM), the glucose was totally depleted before reaching the biofilm bottom (shown by the appearance of inactive cells). Thus, the metabolism of cells located at the intermediary and bottom layers of the biofilm was controlled by the glucose scarcity. This corroborates the hypothesis that the crowding effect is only visible under nutrients limitations.

With a low glucose concentration of 1.1 mM, the biomass and acetate production in the system was considerably reduced under all assumptions. As in the previous glucose concentrations, zones with respiration and fermentation phenotypes, as well as inactive cells were detected in the system under all Cs. Additionally, an acetate-consuming phenotype (acetate + glucose + O_2_ ➔ biomass) was identified in C2-4 simulations (Fig 4), where the acetate locally secreted by cells with respiration and fermentation phenotypes is consumed by acetate-consuming cells. This indicates that the crowding assumption used in the modeling of microbial systems not only affects the time when a phenotype appears but also the emergence of cross-feeding interacions among cells (in this case represented by the acetate exchange between respiration/fermentation cells and acetate consumers). As mentioned above, the simultaneous uptake of acetate and glucose is the result of the optimum proteome allocation of the cell under the local environmental conditions. However, the cell adaptation to new conditions can be a long process involving the gene expression and the synthesis of enzymes (not considered in our simulations). The expression problem can further be integrated in spatio-temporal simulations by using methodologies such as expression and thermodynamics-enabled flux models (ETFL) [36] into CROMICS. The emergence of phenotypes depends on several factors, such as the presence of two or more carbon sources, the protein expression level, etc. For example, the consumption of lactose by *E*. *coli* is determined by the expression of the *lac* operon, which is regulated by the glucose concentration in the medium. When the gene expression depends on the availability of a particular substrate, crowding conditions could modify the time required by microorganisms to adapt to new environmental conditions (i.e. lag phase), and therefore the time (and may be the spatial location) at which the new phenotype appears. In this sense, the selection of an appropriate crowding assumption becomes highly relevant for modeling microbial communities with cross-feeding interactions, because the synthesis of a cross-feeding metabolite can be associated to especific phenotypes.

### Effect of the microbial growth conditions: planktonic vs biofilm culture

The environmental conditions determine the living state of the microbial community—either in a planktonic, free-floating mode or as a biofilm. In biofilms, the matrix of extracellular polymeric substances (EPS) functions to keep the cells attached to a surface and protect the microorganisms from flow shear forces. In such space-constrained systems, cellular motion is limited, and the expansion of the community occurs through cell-shoving mechanisms. In a free-swimming state, however, cells can randomly move across the system (Brownian motion) or even bias their motion towards nutrient-rich regions (chemotaxis). Therefore, cells are more easily redistributed in a planktonic-type system. Here, we wanted to analyze the influence of crowding conditions on both planktonic and biofilm growth modes.

For comparison purposes, we used the same base case study to simulate the microbial growth of *E*. *coli* in both of cultures, differing only in that cells in the planktonic mode were randomly distributed through the whole system and diffused by Brownian motion. The probability of cell motion was determined by the diffusion coefficient of non-motile *E*. *coli* (*D*_*sp*_), i.e., *P*_*sp*_ = *n*_*vox*_*D*_*sp*_Δ*t*/Δ*x*^2^.

Because cells in their planktonic state can move around, it is easier for the system to maintain a smaller number of individuals in the different regions such that there are less crowded zones than in a biofilm. Such non-crowded or “diluted” conditions dominated the first four hours of our planktonic simulation, where there was only a negligible crowding influence on the biomass and acetate production. This means that C1, C2, and C4 had similar results during the first hours, when cells occupied less than 14% of the total volume of the system. Nevertheless, as the system became more crowded due to the increasing number of cells (i.e. the volume occupied was greater than 14%), the biomass and acetate predicted at *t* = 8.5h by C2 and C4 deviated from the C1 estimates (Planktonic-SqL in Fig 3A and 3B). As we saw for the biofilm simulation that used 2.25 mM of glucose, the differences in the planktonic microbial growth under the four Cs were due to the ease of nutrient distribution in the systems. In other words, the local availability of the metabolites depends on the free fraction volume, which both delays the appearance of the different phenotypes and affects their abundance in the system (Planktonic-SqL in Fig 3C–3E).

While a similar trend in the dynamics of the system were found under the different Cs, there were also differences noted between planktonic cells and biofilms. For instance, a greater number of cells with respiration phenotype were identified in the planktonic culture (Planktonic-SqL in Fig 3C) as compared to the biofilm (Biofilm-SqL in Fig 3C). This is because a greater number of cells can access regions richer in nutrients, i.e., those located near the nutrient source (top of the system). Therefore, the planktonic cells grow faster than when in a biofilm. Since the population grew faster in the planktonic state, the nutrient depletion rate was also greater, and inactive cells appeared 6 min earlier than in the biofilm for C1.

In bioreactors, the well mixing is essential to guarantee the optimal production yield. The perturbations of the species metabolism originated by the spatial differences in the nutrient availability can cause the synthesis of undesirable by-products. Even more, as shown in our simulations, the crowding effect on the microbial behavior becomes important as soon as the biomass starts to accumulate in a region occupying more than 14% of the volume. An example of where crowding conditions would need to be considered in the modeling of microbial planktonic communities is the growth of *Staphylococcus aureus* in liquid cultures, which forms cellular aggregates of 60–80 μm in diameter. These planktonic aggregates provide cells with a higher metabolic activity than those in a biofilm, and also offer protection to the community against antimicrobial treatments [37].

### Effect of the grid geometry: Hexagonal vs square lattice

The cell size and the arrangement in which the cells are organized/distributed in the system could affect the available volume for nutrient diffusion. For instance, the secretion of EPS molecules increases the spacing between cells, which could reduce the local crowding conditions. In this section, we analyze how cell packing affects microbial growth in a biofilm. Two cell arrangements were tested: hexagonal and squared. In a hexagonal arrangement, spherical cells can occupy up to 60.4% of the space, while in a squared lattice, the densest packing is 52.3%.

For comparison purposes, the system defined in our base case study was divided into 100 x 100 boxes of a regular shape that can contain only one cell. For the hexagonal lattice (HexL), a box was represented by a hexagonal prism of Δ*x* = 1.1 x 10^−3^ mm per side and 1.3 x 10^−3^ mm in height, whereas in the square lattice (SqL), the boxes were small cubes of Δ*x* = 1.3 x 10^−3^ mm per side. Biofilm simulations on both types of lattice configurations and C1, showed that the amount of biomass and acetate predicted in HexL was 40% and 37% of the values computed for SqL (S3 Fig). Additionally, although the cells grew slowly in HexL, the fraction of active cells (respiration and fermentation phenotypes) present at the end of the simulation was slightly lower than in SqL (43% and 50%, respectively, Fig 3C and 3D). However, the inactive cells appeared in the HexL system 1.6 h before SqL (Fig 3E).

This apparent influence of the crowding conditions on the biomass produced and the number of active cells in the system can be explained by the activity coefficient *γ* of the nutrients in both HexL and SqL. As mentioned in the section Effect of the crowding assumptions, *γ*_*met*_ determines (i) effective diffusion (Eq 25), and (ii) the effective concentration of nutrients (Eq 15). When a cell (with radius *R*_*max*,*cell*_) occupies the maximum volume fraction allowed in a HexL-box, the activity coefficient of glucose (Eq 16 for *R*_*glc*_ = 0) is estimated as *γ*_*glc*_ = 1/(1–0.604) = 2.52, while in a SqL-box, *γ*_*glc*_ = 1/(1–0.523) = 2.09. Under such conditions, and assuming a glucose concentration *C*_*glc*_ of 0.01 mM in both HexL and SqL boxes, aTFA predicts that the cell in HexL grows 0.01 h^-1^ faster and produces 0.34 mmol gDW^-1^ h^-1^ more acetate than in SqL. Therefore, HexL systems are more sensitive to changes in the crowding conditions, e.g. those originated by cell-size increments, than SqL configurations. On one hand, the microbial growth decreases in HexL as a result of the reduced diffusion of metabolites, and thus, the local availability of the nutrients. On the other hand, the metabolic activity of the cells is enhanced due to the increase in the effective concentration of the nutrients.

Comparing the results computed under the different crowding assumptions in HexL, C2 and 4 predict higher biomass and acetate values compared to C1, while C3 underestimates these values (Biofilm-HexL in Fig 3A and 3B). Although a similar trend was found in SqL, some differences were predicted for the growth and phenotypic diversity in HexL simulations when the different crowding assumptions were applied (Fig 3). For example, inactive cells also appeared in the system almost immediately using C3 in HexL, while in SqL, this phenotype only appears after 42 min (Fig 3E). Since biofilm in HexL grew slowly reaching a height of 0.04 ± 0.0068 mm in C1, while in SqL the height was 0.0917 ± 0.012 mm, cells were exposed to different nutrient concentration in both HexL and SqL biofilms. To fairly assess the impact of lattice configuration on the number of active cells, biofilms were compared at the time they reached an average height of 0.04 mm under C1, that is after 3.5 h in SqL and 8.5 h in HexL. The number of active cells (respiration and fermentation phenotypes) estimated by C1 in HexL was 22% less than those predicted for C4, while in the SqL simulations, the difference was 7%. These results indicate that the higher packing density achieved in HexL potentiates the crowding effect on the diffusion and concentration of the nutrients in the system, and therefore, that HexL simulations are more sensitive to the crowding assumption selected. In our 2D simulations, cells can occupied up to 52.3% and 60.4% of the space in SqL and HexL respectively, however, in 3D systems the densest packing volume of spheres could reaches 74% (corresponding to close-packed face-centered cubic lattice). A maximum value of 64–65% has been used to simulate the grow of bacterial colonies [3,38]. Since 3D systems can achieve denser packaging values, the crowding effect may be more pronounced in such systems.

Frequently, microbial systems are composed of cells of different species, each one characterized by a cell size and shape. Along with other factors, such as the production of EPS molecules, the cell size and shape determine the closest packing order of a system. Thus, a multispecies biofilm can consist of regions with different packing orders, which may give a competitive advantage to the species located with more free space between the individuals and/or components, favoring nutrient penetration into the biofilm.

## Conclusion

Crowding conditions can reduce the effective diffusion and enhance the effective concentration of the metabolites. The crowding assumption made in microbial simulations could over/underestimate the nutrients availability and cause errors in the prediction of microbial growth, synthesis of by-products, and even the emergence of new phenotypes. We found that the crowding effect should be taken into account when cells occupy more than 14% of the local volume fraction, either because the system has close packing structures (e.g. biofilm), or when biomass accumulates in a region due to microbial adhesion mechanisms or the lack of well-mixing. Only high nutrient concentrations can dampen the crowding effect, so it can be safely neglected (using assumption C4) when rich-nutrient environments are simulated.

For poor-nutrient conditions or microbial systems with cross-feeding interactions where exchange metabolites are in low concentrations, more realistic results can be obtained by applying a reduced effective diffusion *D*_*eff*_ = *xD* only in boxes containing biomass (as in assumptions C1 and C2). An average *x* value can be obtained experimentally or from detailed crowding simulations. The use of a more detailed representation of local crowding conditions (taking into account for example the size and abundance of cells and other macromolecules in the medium, as was done in C1) is more suitable for multispecies systems, where species can form heterogeneous cellular arrangements or close packing structures. These considerations suggest that detailed crowding modeling can shed light on the effect of the disruption of biofilm architecture on the cell metabolism, where species may repond differently to the chemical treatment, e.g. secreting enzymes to deactivate antibiotics that also modify the local crowding conditions.

## Methods

### The CROMICS methodology

**Fig 5:**
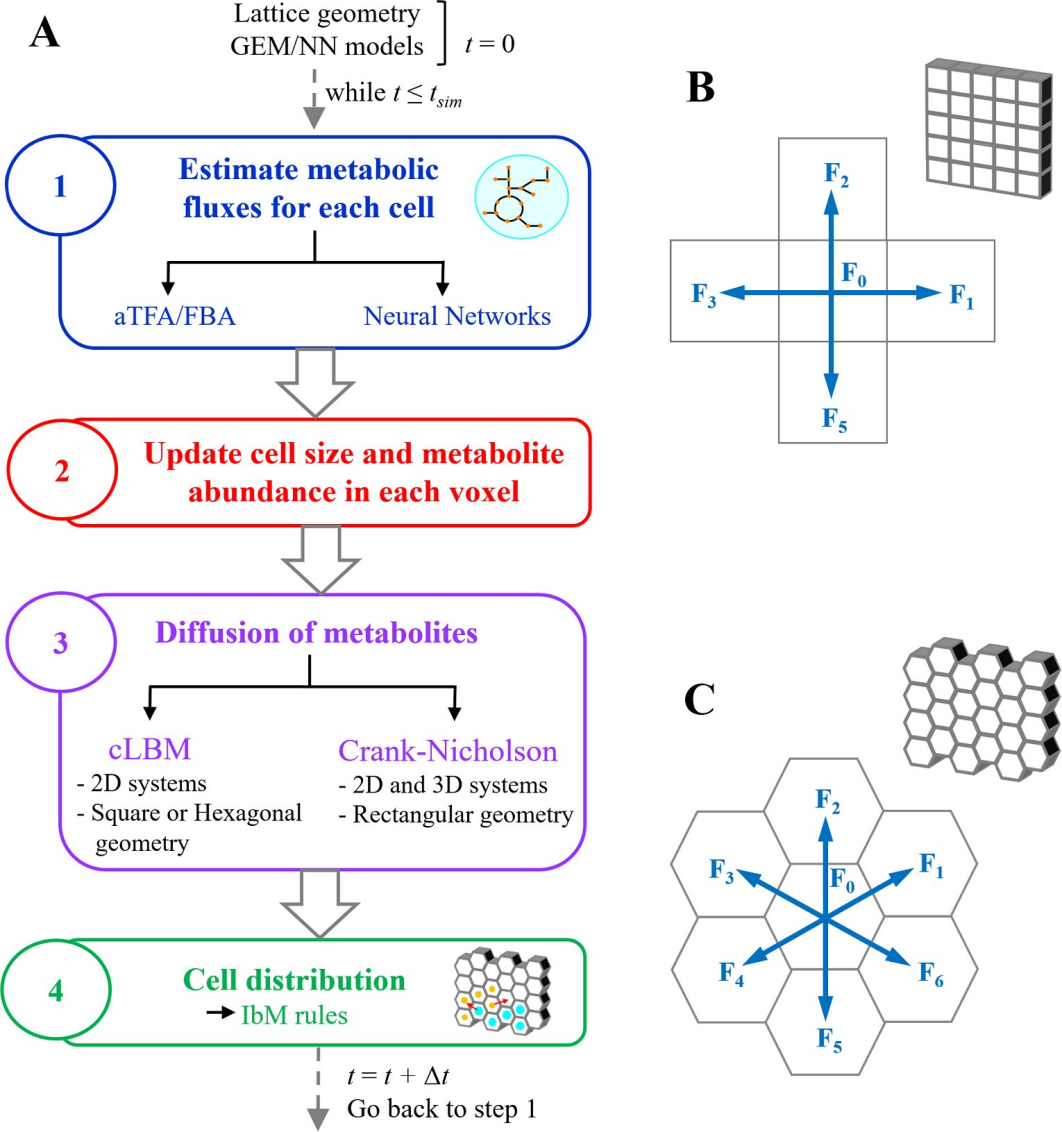
Workflow of CROMICS. (A) At every time step Δ*t*, the metabolic fluxes of each cell in the system are estimated using either (an allocation version) thermodynamic flux analysis (TFA) or neural networks. Then, the fluxes estimated are used to update the cells size and metabolite abundance in the boxes. Metabolites are allowed to diffuse to neighboring boxes, this step can be computed by crowding lattice Boltzmann method (cLBM) or the Crank-Nicholson approximation. Finally, the spatial distributions of cells is computed using individual-based model (IbM) rules. Either a square (B) or a hexagonal lattice (C) can be selected for the 2D simulation of clustered microbial system. Blue arrows indicate the lattice directions, indicating the motion direction, for both cLBM and IbM approaches.

Recently, we developed CROMICS [29], an IbM model that computes the metabolic response of individual cells to local environmental conditions in heterogeneous systems. For this purpose, the total simulation time *t*_*sim*_ is divided in time steps Δ*t*, while the system is discretized into regular boxes, which contain nutrients and other solid components, such as EPS molecules. To study the effect of the cellular arrangement or grid geometry on the microbial simulation, we adapted CROMICS to test two lattice geometries in 2D systems: square and hexagonal. Thus, the 2D system is represented by a monolayer of either hexagonal- or cubic-prism boxes. cLBM and IbM are lattice-on methodologies, so they both use the same lattice geometry in the simulations. Here, we assume that a box of side Δ*x* can contain at most one spherical whose maximum cell radius (just before cell division) is *R*_*max*,*cell*_ (mm). Thus, the box volume *V*_*box*_ (mm^3^) is equal to ${8R}_{\max,\mathrm{cell}}^{3}$ and ${4\surd 3R}_{\max,\mathrm{cell}}^{3}$ for a square or hexagonal lattice, respectively. The (iterative) CROMICS methodology (Fig 5A) can be summarized as follows at every time step Δ*t*:

1. Compute the metabolic fluxes of each cell in the system. During a time Δ*t*, each cell metabolizes the nutrients located at the same box *ij*, synthesizes and releases some by-products to the medium, and the cell increases in size. From the stoichiometric information of the metabolic pathways, the growth rate *v*_*bio*_ as well as the uptake/production rate *v*_*f*,*ex*_ are calculated using aTFA. Alternatively, NNs can be trained and used to approximate the flux solutions *v*_*bio*_ and *v*_*f*,*ex*_ obtained by aTFA. See details in the section entitled Estimation of the metabolic fluxes using aTFA.
2. The mass, radius, and phenotype of each cell as well as the number of metabolites present in each box *ij* are updated using the metabolic fluxes *v*_*bio*_ and *v*_*f*,*ex*_ computed in step 1. Table 3 summarized the complementary equations used to update the microbial cells and metabolite information for the CROMICS simulations.
3. Compute the diffusion of the metabolites present in the medium. Since the crowding conditions in each box change due to cell growth, the probability of the metabolites finding available space in the neighboring boxes decreases, and therefore, the motion of the molecules is also reduced. In this paper, we use cLBM to simulate the nutrient motion in the system (see details below).
4. Redistribution of the microbial cells in the system. Cell division and death, as well as the motion of the cells across the lattice, are simulated by IbM rules that determine the new spatial distribution of the individual cells. Thus, cell division takes place when cell mass *M*_*cell*_ reaches the maximum dry mass *M*_*max*,*sp*_. The daughter cell is allocated in one (empty) neighboring box. If no empty sites are found, then the daughter cell will *shove* the others until all cells are allocated in different boxes. Conversely, when *M*_*cell*_ ≤ *M*_*min*,*sp*_ (the minimal dry mass of a cell), the cell will die and disappear from the lattice. If cells are growing in a liquid medium in a planktonic state, the random motion of a cell can be simulated using the Monte Carlo (MC) algorithm.
5. Update the time *t* = *t* + Δ*t*, and return to step 1.

**Table 3:** Complementary equations used by CROMICS for the spatio-temporal simulation of microbial systems.

Equation	No.	Notes
Upper limit of the uptake flux $v_{f,ex,met}^{U}=\frac{V_{M,met}C_{eff,met}(ij,t)}{K_{M,met}+C_{eff,met}(ij,t)}$ $v_{f,ex,met}^{U}=\min\left(V_{M,met},\rho_{met}(ij,t)/M_{cell}(t)\Delta t\right)$	(13) (14)	Active transport, given by the Michaelis-Menten equation. Passive transport, e.g. O_2_ and acetate.
Effective concentration of metabolites $C_{eff,met}(ij,t)=\frac{\rho_{met}(ij,t)}{10^{6}V_{box}}\gamma_{met}(ij,t)$	(15)	The factor 10^-6^ converts units of mm^3^ into L. $\frac{\rho_{met}(ij,t)}{10^{6}V_{box}}$ represents the concentration in a box.
Activity coefficient of the metabolites scaled particle theory (SPT) [14,15] $\ln\gamma_{met}=-\ln(1-S_{3})+(\frac{6S_{2}}{1-S_{3}})R_{met}+(\frac{12S_{1}}{1-S_{3}}+\frac{18S_{2}^{2}}{{\left(1-S_{3}\right)}^{2}})R_{met}^{2}+\left(\frac{8S_{0}}{1-S_{3}}+\frac{24S_{1}S_{2}}{{\left(1-S_{3}\right)}^{2}}+\frac{24S_{2}^{3}}{{\left(1-S_{3}\right)}^{3}}\right)R_{met}^{3}$ *S*_*x*_ (*x* = 1,2,3) is given by $S_{x}=\frac{\pi }{6V_{box}}\left(\sum_{l}^{macromolecules}\frac{\rho_{l}N_{A}}{10^{3}}{\left(2R_{l}\right)}^{x}+\Phi \right)$ Where Φ for cell-permeable metabolites (e.g. O_2_) is $\Phi =\sum_{l}^{cells}{\frac{M_{cell}N_{A}}{{MW}_{protein}}(2R_{protein})}^{x}$ Φ for not cell-permeable metabolites $\Phi =\sum_{l}^{cells}{\left(2R_{cell}\right)}^{x}$	(17A) (17B) (18) (19)	The box index *(ij*,*t)* has been dropped from *γ*_*met*_ and *S*_*x*_. In *S*_*x*_, 10^-3^ is the conversion factor from mol to mmol. *γ*_*met*_ ∝ *R*_*protein*_ of the intracellular proteins of 72 kDa [22]. *M*_*cell*_*N*_*A*_*/MW*_*protein*_ is the number of proteins in a cell.
Update the number of metabolites in each box $\rho_{met}(ij,t+\Delta t)=v_{f,ex,met}(ij,t)M_{cell}(ij,t)\Delta t+\rho_{met}(ij,t)$	(20)	
Update the mass, radius, and phenotype of each cell $M_{cell}(ij,t+\Delta t)=v_{bio}(ij,t)M_{cell}(ij,t)\Delta t+M_{cell}(ij,t)$ $R_{cell}(ij,t+\Delta t)={\left(\frac{3}{4\pi }M_{cell}(ij,t+\Delta t)\upsilon_{sp}\right)}^{1/3}$ $Phenotype=\left\{ \begin{array}{cc} 1 & if\;v_{f,ex,met}\geq \theta \\ 2 & f\;v_{f,ex,met}<\theta \end{array}\right.$	(21) (22) (23)	The threshold value *θ* was set as 10^-4^ mmol g_DW_^-1^ h^-1^.
Radius of the metabolites or proteins *R_met_* = (3*MW_met_υ_met_*/4*πN_A_*)^1/3^	(24)	
Effective diffusion coefficient *D_eff,met_*(*ij,t*) = *D_met_γ_met_*(*ij,t*)^−1^	(25)	

Nomenclature: *C*_*eff*,*met*_, effective concentration (mmol L^-1^); *D*_*met*_, diffusion in water; *D*_*eff*_, effective diffusion coefficient; *k*, Boltzmann constant (Pa mm^3^ K^-1^); *K*_*M*,*met*_, Michaelis-Menten constant (mmol L^-1^); *M*_*cell*_, cell mass (g_DW_); *MW*_*met*_, *MW*_*protein*_, molecular weight of the metabolite *met* or intracellular proteins (g mol^-1^); *N*_*A*_, Avogadro constant (molecules mol^-1^); *Phenotype*, phenotype of the cell (dimensionless); *R*_*cell*_, *R*_*met*_, *R*_*protein*_, radii of the cell, metabolites, or proteins (mm); *ν*_*bio*_, growth rate (h^-1^); *v*_*f*,*ex*,*met*_, exchange flux of metabolite *met* to/from cells (mmol g_DW_^-1^ h^-1^); *v*^*U*^_*f*,*ex*,*met*_, upper limit of the metabolic exchange flux (mmol g_DW_^-1^ h^-1^); *V*_*M*,*met*_, physiological maximal uptake flux (mmol g_DW_^-1^ h^-1^); *V*_*box*_, box volume (mm^3^); *γ*_*met*_, activity coefficient of metabolite *met* (dimensionless); *ρ*_*met*_, amount of metabolite *met* (mmol); *υ*_*sp*_, *υ*_*met*,_ specific volume constant for the microbial species *sp* or metabolite *met* (mm^3^ g^-1^); Δ*t*, simulation time step (ms).

CROMICS is fully described in [29]. In this paper, we present only the new methods and techniques incorporated into CROMICS.

### Estimation of the metabolic fluxes using aTFA

The local availability of nutrients and the metabolic capabilities of the microbial species determine the metabolic flux distribution inside a cell and the maximum growth rate *ν*_*bio*_ attained in the box *ij*. To estimate the metabolic fluxes, we implemented aTFA, a proteome allocation constrained version of thermodynamics flux analysis [8]. aTFA is a mixed-integer linear problem (MILP) seeking to maximize *ν*_*bio*_ (h^-1^), subject to mass conservation, thermodynamics [13], and proteome allocation constraints [16], i.e.,

$$\max\;v_{bio}$$ (1)

s.t.N∙v (2)

$$0\leq v_{f}\leq {z_{f}v}_{f}^{U}$$ (3)

$$\Delta_{r}{G^{\prime }}_{f}-Q+Qz_{f}<0$$ (4)

$$\Delta_{r}{G^{\prime }}_{f}-RT\sum_{a=1}^{m}\eta_{f,a}c_{a}-\Delta_{r}G_{f}^{\prime o}<0$$ (5)

$$c_{a}^{L}\leq c_{a}\leq c_{a}^{U}$$ (6)

$$\sum_{ex}w_{ex}v_{f,ex}+\sum_{u}w_{u}v_{f,u}+w_{R}v_{bio}=\phi_{max}$$ (7)

**N** is the [*m* x *n*] stoichiometric matrix of the *m* intracellular metabolites and *n* biochemical and transport reactions, and **v** is the [*n* x 1] flux vector. As in the original TFA formulation, each reversible reaction (and the corresponding flux) in this formulation is divided into its forward and backward components. Eq 3 restricts the maximum flux value *v*^*U*^_*f*_ (mmol g_DW_^-1^ h^-1^) that a reaction *f* can take for the current environmental conditions. The exchange flux *v*^*U*^_*f*,*ex*,*met*_ is determined by the local substrate concentration *met* and the active/passive transport inside the cell (Eq 13 and 14 in Table 1).

The binary variable *z*_*f*_ (*z*_*f*_ = 1 when *v*_*f*_ > 1, otherwise *z*_*f*_ = 0) couples the thermodynamic and mass constraints by preventing the flux through a reaction with positive Gibbs free energy ${\Delta_{r}G}_{f}^{\prime }$ (Eqs 3 and 4). In Eq 4, *Q* is a constant with a large value (arbitrary). The value of ${\Delta_{r}G}_{f}^{\prime }$ is given by the stoichiometric coefficient *η*_*f*,*a*_ and the logarithm of the intracellular concentration *C*_*int*,*a*_ of the metabolites *a* involved in reaction *f*, i.e., *c*_*a*_ = ln(*C*_*int*,*a*_/*C*_*0*_) where the standard concentration *C*_*0*_ is equal to 1 M (Eq 5). In Eq 5, *R* is the ideal gas constant, *T* is the temperature, and ${\Delta_{r}G}_{f}^{\prime o}$ is the standard Gibbs free energy, which was estimated by the group contribution method [39]. The limits *c*^*L*^_*a*_ and *c*^*U*^_*a*_ were set to -13.81 and -2.99, respectively [13].

Finally, Eq 7 constraints the way the proteome fraction available in the cell, *ϕ*_*max*_, is divided to carried out metabolic processes. Thus, a protein cost *w* is associated with each flux *f* that depends on the type of processes that flux represents, e.g. the exchange/transport of metabolites (identified with the suffix *ex*), enzymatic reactions (with suffix *u*), and ribosomal proteins required to maintain a growth rate (with suffix *R*). The weighting parameters are set to *w*_*u*_ = 1.55 x 10^−3^ g_DW_ h mmol^-1^, *w*_*R*_ = 0.169 h [16], and by assuming that the substrate uptake rate is only limited by the substrate availability in the box (see below), then *w*_*ex*_ = 0.

CROMICS uses neural networks (NNs) as an alternative for estimating *v*_*bio*_ and *v*_*f*,*ex*,*met*_. The advantage of using NNs is the fast computational time for the exchange metabolic fluxes, even when complex objective functions are required by the stoichiometric-based models. The NN of a microbial species is trained using (as outputs) the metabolic flux solutions computed by aTFA for 100,000 uptake-flux samples (inputs). These flux samples were used to constrain the upper flux limits (*v*^*U*^_*f*,*ex*,*met*_) of the corresponding metabolites in the aTFA formulation (Eq 1c). An uptake-flux sample consists of *n* uptake fluxes, such as glucose, acetate, and O_2_ (as in our case study), where the flux of each metabolite *met* was randomly taken from the range 0 to *V*_*M*,*met*_, i.e., the physiological maximum uptake flux (mmol g_DW_^-1^ h^-1^). Other exchange fluxes in the GEM model were left unconstrained (i.e. *v*^*U*^_*f*,*ex*,*met*_ = 1000 mmol g_DW_^-1^ h^-1^). To reduce the possible alternative flux solutions originating from underdetermined stoichiometric systems, two consecutive aTFA optimizations—the maximization of biomass and the subsequent minimization of the sum of the fluxes subject to previously computed *v*_*bio*_ [40]—were performed to generate the training data for the NN. If no feasible solution was predicted by aTFA due to the depletion of nutrients, then the cell was allowed to shrink at a rate of *v*_*shrinkage*_ in an attempt to maintain the cell energy requirements.

For *E*. *coli*, a NN was created containing 2 hidden layers of 15 neurons each using the Neural Network toolbox of Matlab R2018b. The Pearson correlation coefficient r between the fluxes predicted by NN and aTFA was estimated to be 0.999, while the normalized mean squared error was 8.5 x 10^−5^ (S4 Fig).

### Nutrient diffusion estimated using cLBM

Unlike the original formulation, where the diffusion of the metabolites is approximated using a Crank-Nicholson scheme [29], in this paper, the motion of the metabolites across the system is simulated using cLBM [17]. cLBM is a lattice Boltzmann approach that allows the simulation of the diffusion of molecules in a 2D crowded media using a square lattice configuration. Among the advantages of cLBM are its easy implementation, fast simulations due to the mesoscopic nature of the method, easy incorporation of the complex geometries of porous medium, and the incorporation of the effect of molecular size on the diffusion. In this paper, we extended cLBM to simulate diffusion in a 2D hexagonal lattice (identified as HexL). The equations below are also valid for both HexL and the square lattice (identified as SqL) when the appropriate LBM parameter/constant is considered in the simulations, as described below.

In cLBM, the system is discretized into boxes of volume *V*_*box*_ that are separated by a distance Δ*x* (mm), where collections of molecules moving across the lattice are followed at every (discrete) time step Δ*t*. The number of metabolites *met*, *F*_*d*,*met*_ (mmols per box), that jump from box *ij* to one of the *n*_*vox*_ contiguous boxes *ij*_*next*_ (see Fig 5B and 5C) under crowding conditions is given by [17]

$$F_{d,met}({ij}_{next},t+\Delta t)=\left(({1-\omega }_{met})F_{d,met}({ij}_{next},t)+\omega_{met}F_{d,met}^{eq}(ij,t)\right)\frac{1}{\gamma_{met}({ij}_{next},t)},\;d=1,\ldots ,n_{vox}$$ (8A)

$$F_{0,met}(ij,t+\Delta t)=\rho_{met}(ij,t)-\sum_{d}F_{d,met}({ij}_{next},t+\Delta t)$$ (8B)

The factor 1/*γ*_*met*_ in Eq 8A determines the probability of finding available space in the target box *ij*_*next*_ to fit the incoming molecules *met* from *ij*. The activity coefficient *γ*_*met*_ is calculated using the scaled particle theory (SPT) (Eq 16). In this way, the crowding conditions affect both the effective concentration *C*_*eff*,*met*_ (Eq 15) and the movement (diffusion) of metabolites across the system (Eq 8).

In Eq 8A, the equilibrium distribution function $F_{d,met}^{eq}$ is expressed as [41]

$$F_{d,met}^{eq}(ij,t)=\rho_{met}(ij,t)w_{d,}$$ (9)

where *w*_*d*_ is a weight factor that depends on the type of grid and the direction *d* in the scheme lattice (Fig 5B and 5C), such that in SqL, *w*_*d*_ = 0 for *d* = 0 and *w*_*d*_ = 1/4 for *d* = 1,…,4, while in HexL, *w*_*d*_ = 3/9 for *d* = 0 and *w*_*d*_ = 1/9 for *d* = 1,…,6.

The macroscopic density of metabolites *met*, *ρ*_*met*_ (mmol per box), is computed as:

$$\rho_{met}(ij,t)=\sum_{d}F_{d,met}(ij,t).$$ (10)

The relaxation parameter *ω*_*met*_ in Eq 8A indicates the relationship between the simulation parameter Δ*x*, Δ*t*, and the diffusion coefficient in a dilute (non-crowded) medium, such as water, *D*_*w*,*met*_ (mm^2^ s^-1^).


$$\omega_{met}=\frac{2}{1+p_{L}D_{w,met}\frac{\Delta t}{{\Delta x}^{2}}}.$$ (11)

The parameter *p*_*L*_ is equal to 4 for SqL, and 8 for HexL [17,41]. The selection of the parameters Δ*x* and Δ*t*, and therefore of *ω*_*met*_, determine the accuracy of cLBM. Numerical difficulties are found for *ω*_*met*_ > 1, and more accurate simulations are obtained when *ω*_*met*_ approaches 1 [17].

In biofilm modeling, the simulated metabolites can have *D*_*w*,*met*_ of different orders of magnitude. For example, the ratio *D*_*w*,*glucose*_/*D*_*w*,*oxygen*_ is equal to 0.33 [12]. Thus, for a set of Δ*x* and Δ*t*, *ω*_*glucose*_/*ω*_*oxygen*_ = 0.66. This leads to inaccuracies in the diffusion results, since *ω*_*met*_ < 1 for at least one of the metabolites. Furthermore, if *met* is the limiting substrate, the error can be propagated to the calculations of the metabolic capabilities of microbial cell.

To improve the simulation accuracy of multispecies diffusing systems, we proposed a correction to cLBM for *ω*_*met*_ < 1, the details of which are provided in S1 Text. Based on Fick’s first law, the number of molecules *F*_*d*,*met*_ (Eq 8A) that jump from one box to another during a time Δ*t* (where *ω*_*met*_ < 1), can be computed directly from $F_{d,met}^{eq}$ using a scaling factor Δ*t/*Δ*t*_*1*_ as:

$$F_{d,met}\;({ij}_{next},t+\Delta t)=\frac{F_{d,met}^{eq}(ij,t)}{\gamma_{met}({ij}_{next},t)}\frac{\Delta t}{{\Delta t}_{1}}d=1,\ldots ,n_{vox},$$ (12)

where Δ*t*_*1*_ is the time step that makes *ω*_*met*_ = 1. The cLBM correction proposed for *ω*_*met*_ < 1 has been validated using the diffusion simulations computed by the MC algorithm [42]. A comparison of the mean squared displacement predicted by the *ω*-correction shows a very good agreement with those computed by MC (see S1 Text).

## Supporting information

S1 Fig Spatial distribution of the metabolites and phenotypes predicted for the *E*. *coli* biofilm with a glucose supply of 2.25 mM at different times. Crowding assumption C1 was used for the simulations.
(TIF)

S2 Fig Volume fraction occupied by cells in the *E*. *coli* biofilm at different times under crowding assumption C1 and 2. The glucose supply was set to 2.25 mM.
(TIF)

S3 Fig Dynamics of the *E*. *coli* biofilm with a glucose supply of 2.25 mM under different crowding assumptions. 2D microbial system was discretized using a square lattice. (A) Biomass. (B) Acetate produced. (C) Active layer depth *H* normalized by Δ*x*. (D) Number cells identified with phenotype respiration: glucose + O_2_ ➔ (acetate) + biomass, fermentation: glucose ➔ acetate + biomass, and inactive cells.
(TIF)

S4 Fig Parity and residual plots of the metabolic fluxes predicted by the allocation version of thermodynamics flux analysis (aTFA) and the neural network (NN) created for *E*. *coli*. The NN with 2 hidden layers of 15 neurons each was trained using 100 K random samples. The normalized mean square error between the fluxes predicted by aTFA and NN was estimated to be 8.5 x 10^−5^, while the Pearson correlation r is 1. Fluxes *v*_*f*_ are given in mmol g_DW_^-1^ h^-1^, while the growth rate *v*_*bio*_ is in h^-1^.
(TIF)

S1 Text cLBM for the diffusion of multispecies systems with *ω*_*met*_ < 1. (DOCX)
