# Supplementary figures and images for: The influence of the crowding assumptions in biofilm simulations
Source: PLoS Comput Biol. 2021 Jul 22;17(7):e1009158. doi: 10.1371/journal.pcbi.1009158 (PMC8297847; doi:10.1371/journal.pcbi.1009158)

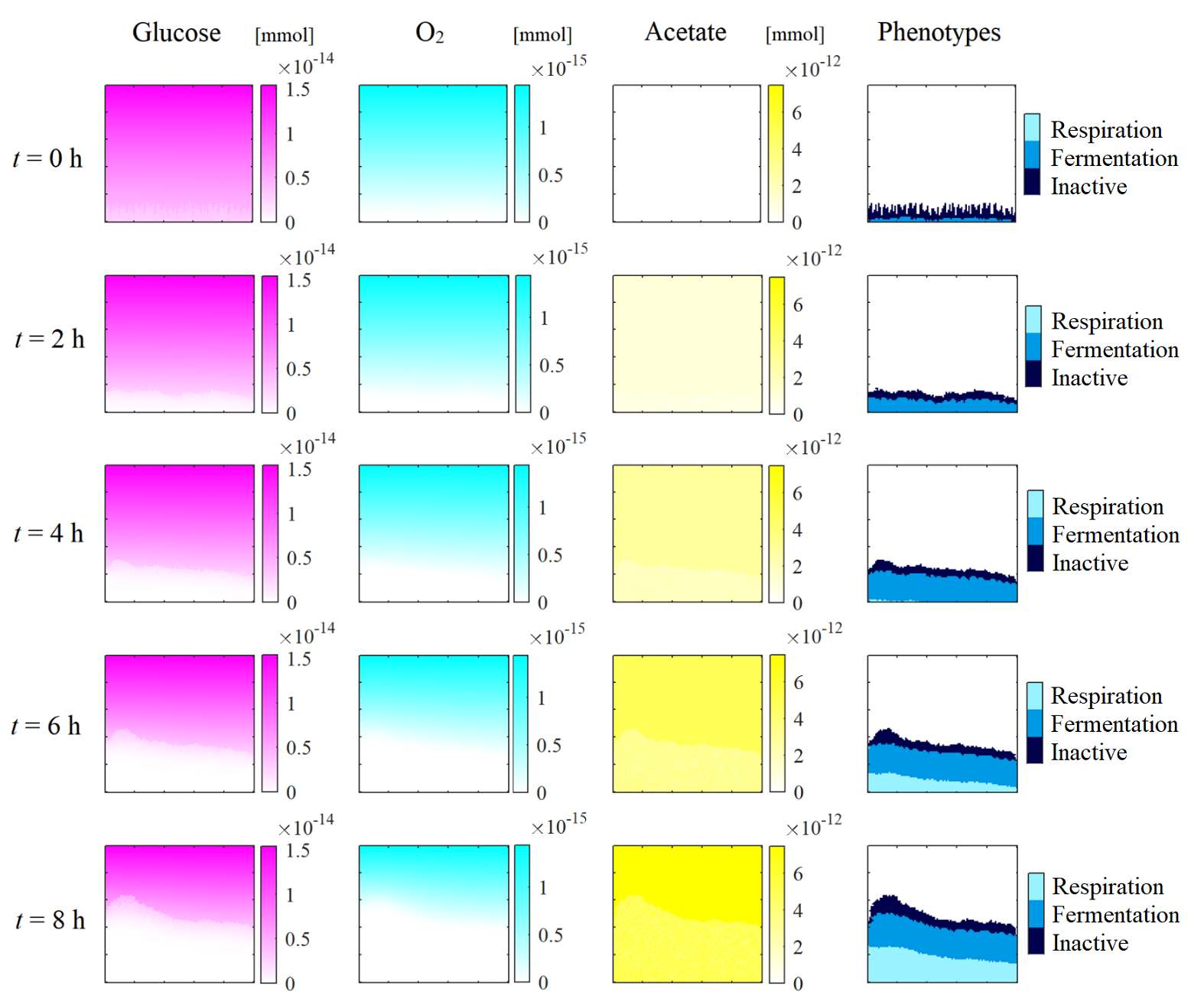

Supplement: S1 Fig — Crowding assumption C1 was used for the simulations. (TIF) [file pcbi.1009158.s001.tif]

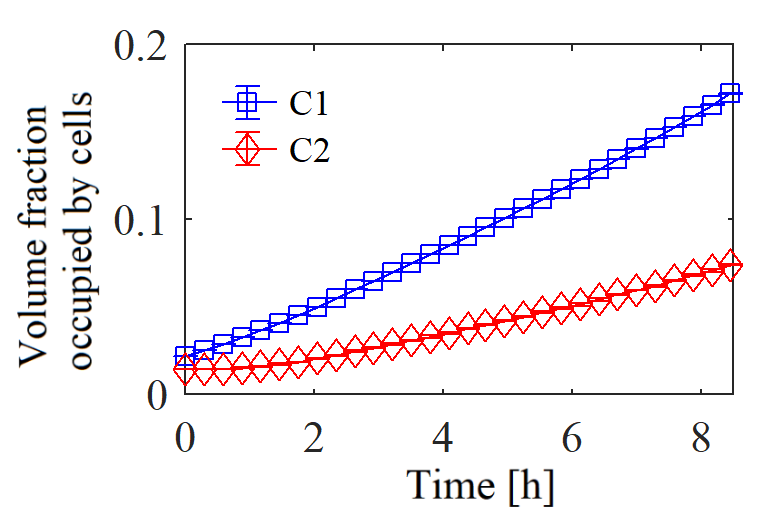

Supplement: S2 Fig — The glucose supply was set to 2.25 mM. (TIF) [file pcbi.1009158.s002.tif]

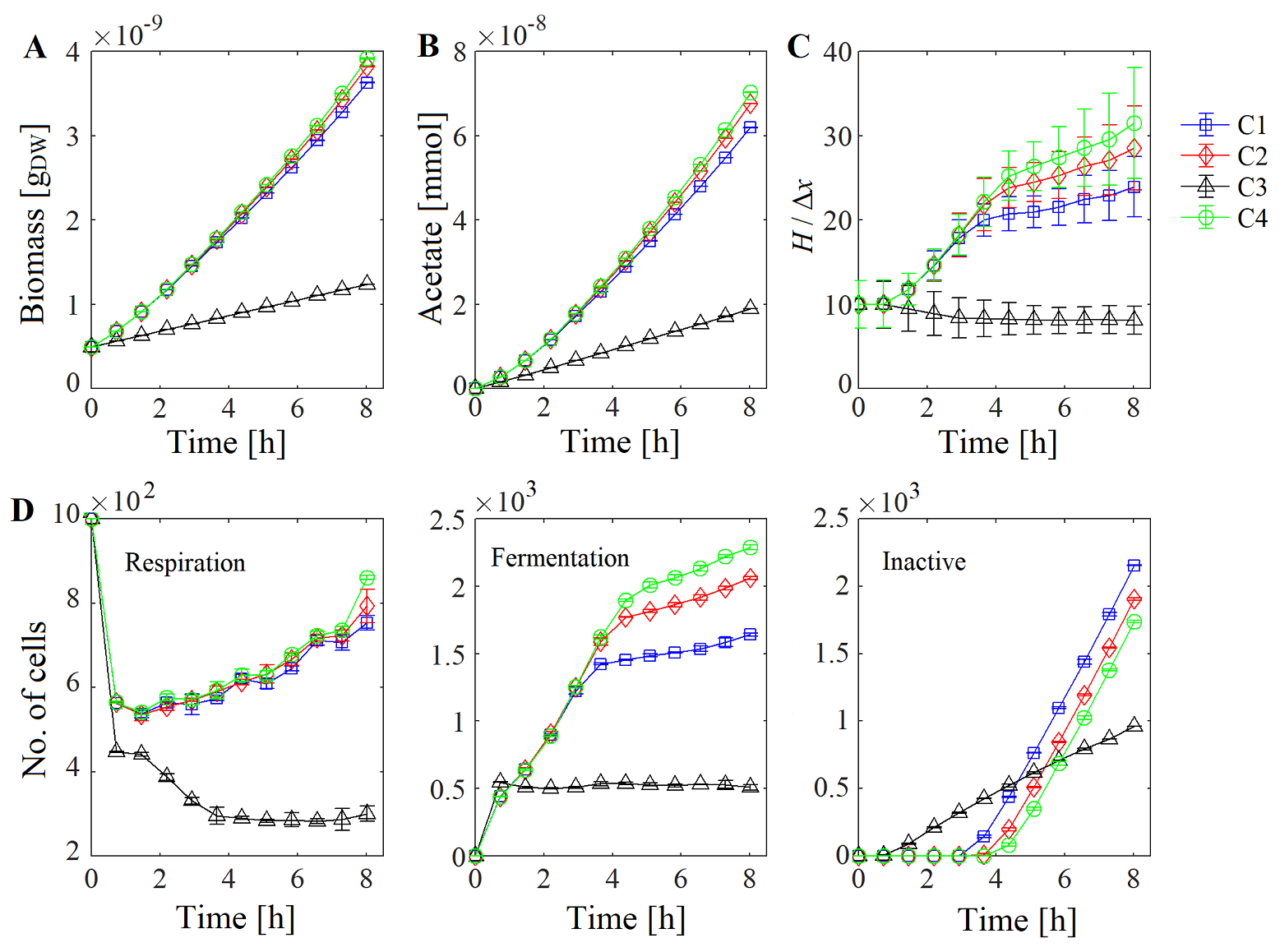

Supplement: S3 Fig — 2D microbial system was discretized using a square lattice. (A) Biomass. (B) Acetate produced. (C) Active layer depth H normalized by Δx. (D) Number cells identified with phenotype respiration: glucose + O2 ➔ (acetate) + biomass, fermentation: glucose ➔ acetate + biomass, and inactive cells. (TIF) [file pcbi.1009158.s003.tif]

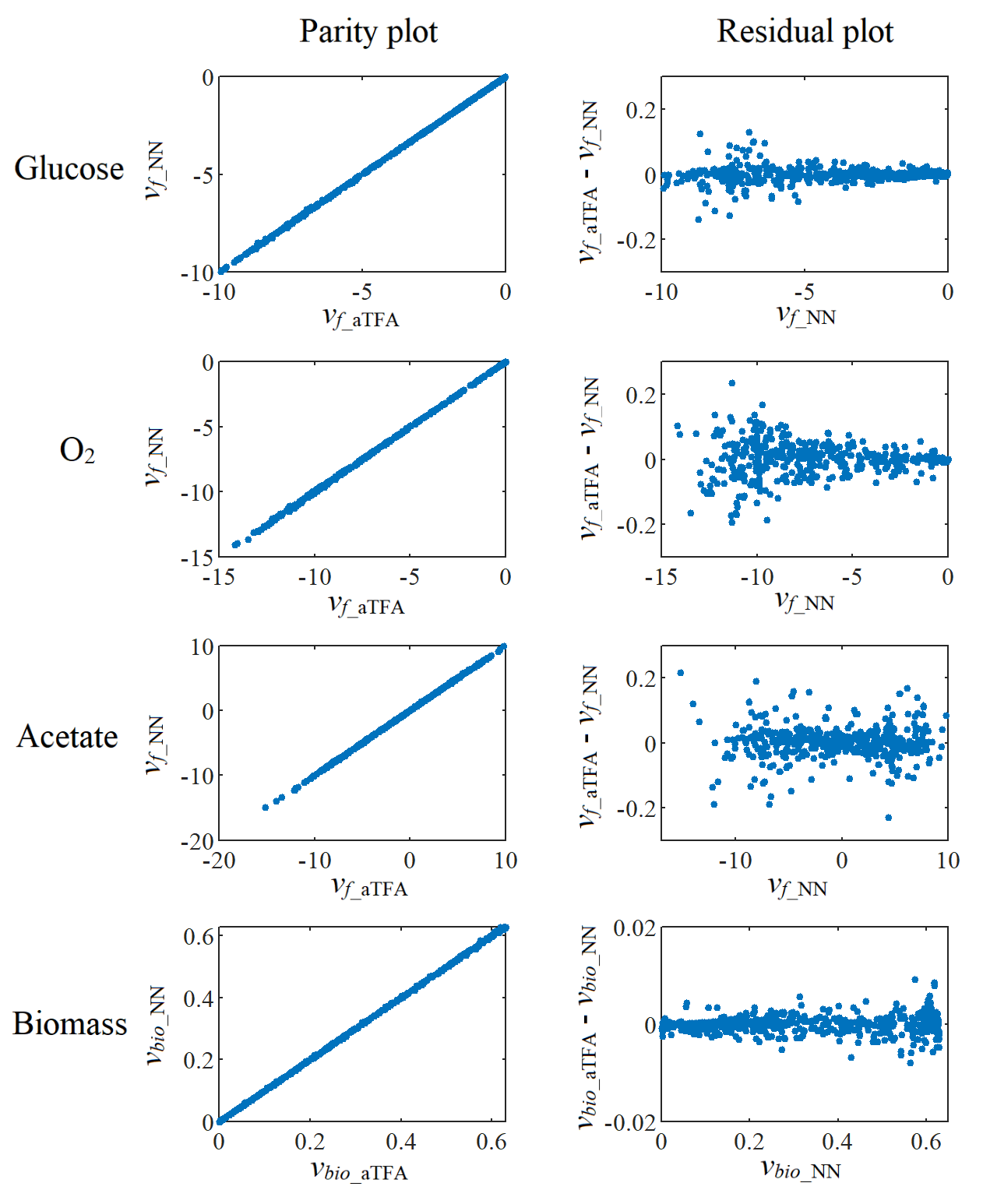

Supplement: S4 Fig — The NN with 2 hidden layers of 15 neurons each was trained using 100 K random samples. The normalized mean square error between the fluxes predicted by aTFA and NN was estimated to be 8.5 x 10^−5^, while the Pearson correlation r is 1. Fluxes vf are given in mmol gDW-1 h-1, while the growth rate vbio is in h-1. (TIF) [file pcbi.1009158.s004.tif]
