# Supplementary material for: The influence of the crowding assumptions in biofilm simulations
Source: PLoS Comput Biol. 2021 Jul 22;17(7):e1009158. doi: 10.1371/journal.pcbi.1009158 (PMC8297847; doi:10.1371/journal.pcbi.1009158)
Supplement: S1 Text — (DOCX) [file pcbi.1009158.s005.docx]

# S1 Text

# The influence of the crowding assumptions in biofilm simulations

Liliana Angeles-Martinez, Vassily Hatzimanikatis

# **cLBM for the diffusion of multispecies systems with  < 1**

The flux *J_met_* of molecules *met* from voxel (*ij*) to (*ij*+1) is determined by the net number of molecules *F* that crossed the normal area Δ*x*^2^ between the two voxels during a time interval $\Delta t_{1}$, i.e.,

$J_{met\left( ij\to ij+1 \right)}=\left( \frac{F_{1,met}^{{\Delta t}_{1}}(ij+1,t+{\Delta t}_{1})}{{\Delta x}^{2}{\Delta t}_{1}}-\frac{F_{3,met}^{{\Delta t}_{1}}(ij,t+{\Delta t}_{1})}{{\Delta x}^{2}{\Delta t}_{1}} \right)\frac{{\Delta x}^{2}}{{\Delta x}^{2}}$. (S1)

$F_{1,met}^{{\Delta t}_{1}}$ and $F_{3,met}^{{\Delta t}_{1}}$ are estimated using a set of parameters Δ*x* and Δ*t_1_*, such that $\omega_{met}^{{\Delta t}_{1}}=1$ (Eq. 11 of the main text). Note that Fick’s first law can be recovered when $\omega_{met}^{{\Delta t}_{1}}=1$ by substituting Eq. 8a, 9, and 11 of the main text in Eq. S1.

Assuming that *J_met_* is constant during Δ*t_1_*, then the amount of *met* per unit of area after Δ*t* (where Δ*t* < Δ*t_1_*) is

$J_{met\left( ij\to ij+1 \right)}\Delta t=\frac{{\Delta x}^{2}}{{\Delta t}_{1}}{\Delta t}_{lbm}\left( \frac{F_{1,met}^{{\Delta t}_{1}}(ij+1,t+{\Delta t}_{1})}{V_{voxel}\Delta x}-\frac{F_{3,met}^{{\Delta t}_{1}}(ij,t+{\Delta t}_{1})}{V_{voxel}\Delta x} \right)$. (S2)

Alternatively, new $F_{1,met}(ij+1,t+\Delta t)$ and $F_{3,met}(ij,t+\Delta t)$ values can be estimated from Eq. 8a of the main text using Δ*x* and Δ*t* as LBM parameters, where *ω_met_* < 1 (Eq. 11 of the main text). Their corresponding $J_{met\left( ij\to ij+1 \right)}\Delta t$ is given by

$J_{met\left( ij\to ij+1 \right)}\Delta t=\frac{{\Delta x}^{2}}{\Delta t}\Delta t\left( \frac{F_{1,met}(ij+1,t+\Delta t)}{V_{voxel}\Delta x}-\frac{F_{3,met}(ij,t+\Delta t)}{V_{voxel}\Delta x} \right).$ (S3)

The aim is to approximate *F_1,met_* and *F_3,met_* (with *ω_met_* < 1) from the more accurate $F_{1,met}^{{\Delta t}_{1}}$ and $F_{3,met}^{{\Delta t}_{1}}$ values (inasmuch as $\omega_{met}^{{\Delta t}_{1}}=1$). For this, both set distribution functions $F_{d,met}^{{\Delta t}_{1}}$ and $F_{1,met}$ must provide the same $J_{met\left( ij\to ij+1 \right)}\Delta t$ value. Thus, *F_1,met_* and *F_3,met_* can be found by comparing Eq. S2 and S3:

$F_{1,met}\left( ij+1,t+\Delta t \right)=\frac{\Delta t}{{\Delta t}_{1}}F_{1,met}^{{\Delta t}_{1}}(ij+1,t+{\Delta t}_{1})$, (S4a)

$F_{3,met}\left( ij,t+\Delta t \right)=\frac{\Delta t}{{\Delta t}_{1}}F_{3,met}^{{\Delta t}_{1}}(ij,t+{\Delta t}_{1})$. (S4b)

Since $\omega_{met}^{{\Delta t}_{1}}=1$, then from Eq. S4a and 11 of the main text, we obtain $F_{1,met}^{{\Delta t}_{1}}\left( ij+1,t+{\Delta t}_{1} \right)=F_{1,met}^{eq}\left( ij,t \right)/\gamma_{met}(ij+1,t)$. Generalizing, Eq. S4 becomes

$F_{d,met} \left( {ij}_{next},t+\Delta t \right)=\frac{F_{d,met}^{eq}\left( ij,t \right)}{\gamma_{met}({ij}_{next},t)}\frac{\Delta t}{{\Delta t}_{1}}, d=1,\ldots,n_{vox.}$ (S5)

Δ*t_1_* is the time step that makes *ω_met_* = 1 (Eq. 11 of the main text), while Δ*t* is the time step used in the LBM simulations, with the constraint that Δ*t* ≤ Δ*t_1_*. Note that if Δ*t* = Δ*t_1_*, Eq. S5 reduces to the classical LBM scheme given by Eq. 8a of the main text.

## **Validation**

To validate the cLBM correction proposed for *ω_met_* < 1 (here identified as cLBM*_ω_*), we compared diffusion simulations computed using Eq. 24 of the main text and the Monte Carlo (MC) algorithm [1,2]. The MC algorithm is a lattice-on technique widely used for the simulation of diffusion and reaction-diffusion systems. Here, time and space are discretized in Δ*t* and voxels of Δ*x* per side. In MC simulations, the non-overlapping restriction given by the impenetrability of the molecules is satisfied by the fact that only one agent (cell or molecule) can be allocated in a voxel.

For a square lattice, the MC can be summarized as follows:

1. Choose a random molecule originally located in voxel (*i*,*j*).

2. Randomly select one of the 4 neighboring voxels.

3. Check that the selected voxel (*i_x_*,*j_x_*) is not occupied by another agent. If so, select a random number $r$ between 0 and 1 and compare *r* with the probability of motion of the agent, i.e., $P=4D\Delta t/\Delta x^{2}$[3], where *D* is the diffusion coefficient. If $r\leq P$, then the agent moves to (*i_x_*,*j_x_*). Otherwise the agent remains in (*i*,*j*).

The diffusion simulation of two types of tracer molecules A and B, each occupying 0.5% of the total lattice space, is carried out on a system of 100x100 voxels of size Δ*x^3^*= 1 nm^3^ and with periodic boundary conditions. The system also contains crowder molecules C that occupy 30% of the total space. We assume that all molecules are non-rotating cubes of size Δ*x^3^*= 1 nm^3^, i.e., only one molecule is allowed per voxel. For this square uniform packing, the activity coefficient *γ* (Eq. 4 of the main text) required by the cLBM simulations can be simplified to $\ln\gamma=-\ln\left(1-S_{3}\right)$ [4]. All molecules are randomly allocated in the system, and their diffusion coefficient is assumed to be $D_{A}=D_{C}=1$ nm^2^ ms^-1^ and $D_{B}=0.333$nm^2^ ms^-1^.

For simulations with a time step Δ*t* = 1 ms, significant deviations were found between the average mean squared displacement (MSD) of molecules A computed by MC (after 100 iterations) and cLBM (without the *ω*-correction). The relative error (Eq. S6) at time 150 ms was estimated to be 73.24% (SA Fig). However, by applying the *ω*-correction in the cLBM simulations, the error decreases to 0.04%. Thus, the *ω*-correction proposed above improves the accuracy of the diffusion simulations in systems where the molecules or agents have diffusion coefficients with different orders of magnitude.

$error_{MSD}(t)=\frac{norm(MSD_{MC}(t)-MSD_{cLBM}(t))}{MSD_{MC}(t)}100$. (S6)


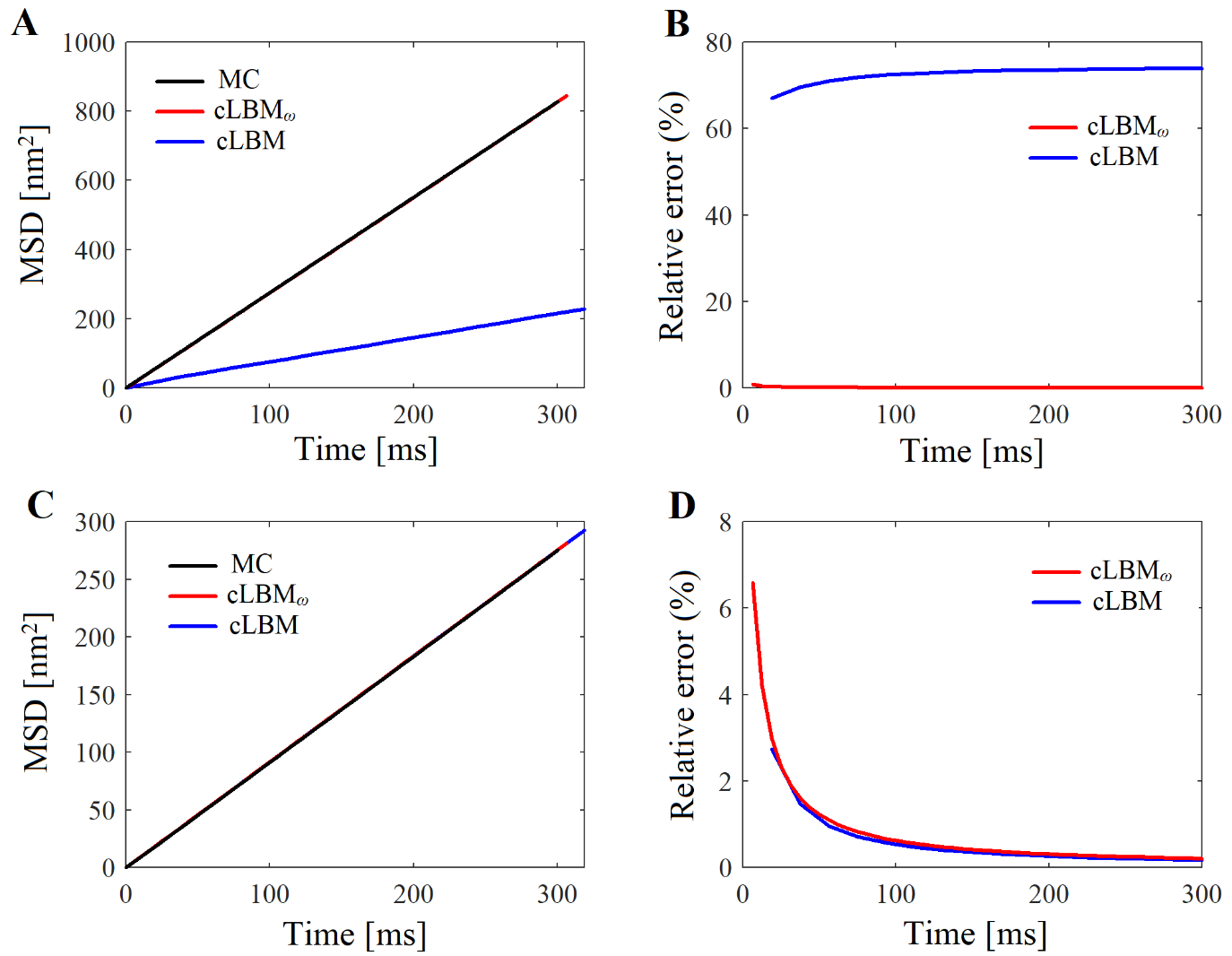


**SA Fig.** **Simulation of the diffusion of molecules A and B in the presence of crowders C that occupy the 30% of the total space**. (A) Mean squared displacement and (B) relative error for molecule A. (C) Mean squared displacement and (D) relative error for molecule B.

References

1. Berry H. Monte Carlo simulations of enzyme reactions in two dimensions: fractal kinetics and spatial segregation. Biophysical Journal. 2002; 83:1891-1901.
2. Saxton MJ. Lateral diffusion in a mixture of mobile and immobile particles. A Monte Carlo study. Biophysical Journal. 1990; 52:1303-6.
3. Ridgway D, Broderick G, Lopez-Campistrous A, Ru’aini M, Winter P, Hamilton M, et al. Coarse-grained molecular simulation of diffusion and reaction kinetics in crowded virtual cytoplasm. Biophysical Journal. 2008; 95: 3748-3759.
4. Angeles-Martinez L, Theodoropoulos C. A lattice Boltzmann scheme for the simulation of diffusion in intracellular crowded systems. BMC Bioinformatics. 2015; 16:353.
